# Insights into the ubiquitin-proteasome system of human embryonic stem cells

**DOI:** 10.1038/s41598-018-22384-9

**Published:** 2018-03-06

**Authors:** Isabel Saez, Seda Koyuncu, Ricardo Gutierrez-Garcia, Christoph Dieterich, David Vilchez

**Affiliations:** 1 Institute for Genetics and Cologne Excellence Cluster for Cellular Stress Responses in Aging-Associated Diseases (CECAD), University of Cologne, Joseph-Stelzmann-Strasse 26, 50931 Cologne, Germany; 2 Department of Internal Medicine III and Klaus Tschira Institute for Computational Cardiology, Section of Bioinformatics and Systems Cardiology, Neuenheimer Feld 669, University Hospital, 69120 Heidelberg, Germany

## Abstract

Human embryonic stem cells (hESCs) exhibit high levels of proteasome activity, an intrinsic characteristic required for their self-renewal, pluripotency and differentiation. However, the mechanisms by which enhanced proteasome activity maintains hESC identity are only partially understood. Besides its essential role for the ability of hESCs to suppress misfolded protein aggregation, we hypothesize that enhanced proteasome activity could also be important to degrade endogenous regulatory factors. Since E3 ubiquitin ligases are responsible for substrate selection, we first define which E3 enzymes are increased in hESCs compared with their differentiated counterparts. Among them, we find HECT-domain E3 ligases such as HERC2 and UBE3A as well as several RING-domain E3s, including UBR7 and RNF181. Systematic characterization of their interactome suggests a link with hESC identity. Moreover, loss of distinct up-regulated E3s triggers significant changes at the transcriptome and proteome level of hESCs. However, these alterations do not dysregulate pluripotency markers and differentiation ability. On the contrary, global proteasome inhibition impairs diverse processes required for hESC identity, including protein synthesis, rRNA maturation, telomere maintenance and glycolytic metabolism. Thus, our data indicate that high proteasome activity is coupled with other determinant biological processes of hESC identity.

## Introduction

Pluripotent stem cells can replicate indefinitely in an undifferentiated state while retaining their potential to differentiate into all cell lineages^1,2^. Embryonic stem cells (ESCs) derived from blastocysts are the gold standard of pluripotency. Moreover, somatic cells can be reprogrammed into induced pluripotent stem cells (iPSCs) that share similar characteristics with ESCs^3,4^. Given their intrinsic abilities, pluripotent stem cells represent an invaluable resource to investigate development and disease, holding great promise for regenerative medicine. As the origin of multicellular organisms, a series of regulatory and quality control mechanisms must operate at high fidelity in these cells^5^. As such, protein homeostasis (proteostasis) is central for self-renewal, pluripotency and cell fate decisions^6–9^.

A key node of the proteostasis network is the ubiquitin proteasome system (UPS), the major selective proteolytic mechanism in eukaryotic cells^10,11^. Notably, human ESCs (hESCs) and iPSCs have increased proteasome activity compared with their differentiated counterparts^12^. This enhanced activity is induced by PSMD11/RPN6^13^, a scaffolding subunit that promotes proteasome assembly^14^. Besides PSMD11, other proteasome regulators (*e.g*., Psmd14, POMP) are up-regulated in mouse and human pluripotent stem cells^6,15,16^. Conversely, a mild down-regulation of proteasome activity induces a demise of hESC identity, characterized by decreased mRNA and protein levels of pluripotency markers^13,15,17^. Concomitantly, pluripotent stem cells with reduced proteasome activity lose their ability to differentiate into neural cells while expressing high levels of markers of endoderm, mesoderm and fibroblast differentiation^13^.

These findings raise the intriguing question of why ESC function needs enhanced proteasome activity. The proteasome terminates damaged, misfolded and aggregated proteins ensued from stress conditions and misfolding-prone mutations^11,18^. The accumulation of damaged proteins could alter the immortality of pluripotent stem cells^5^. Notably, growing evidence indicates that increased proteasome activity prevents the aggregation of damaged proteins such as mutant huntingtin, which underlies Huntington’s disease^9,12,13,19^. Moreover, the passage of altered proteins to progenitor cells could compromise organismal development and aging. In this regard, enhanced proteasome activity participates in the degradation of damaged proteins in the early steps of mouse ESC differentiation, a key step to generate daughter cells with an intact proteome^20,21^.

The UPS also modulates half-life of a myriad of regulatory proteins^22,23^, in a dynamic process adjusted to the specific requirements of a particular cell type and status^24,25^. Since the differentiation process triggers numerous changes in the cellular proteome, the UPS determines successful organismal development^5^. In these lines, the proteasome modulates the abundance of key pluripotency factors such as OCT4 or NANOG^26–29^. A chain of at least four 48-linked ubiquitins is the primary signal for recognition and degradation by the proteasome^30,31^. E3 ligases transfer ubiquitin to a target protein, providing specificity to the proteolytic process. Accordingly, over 600 E3 ubiquitin ligases have been identified in humans so far. To prevent proteolysis, deubiqutinating enzymes (DUBs) perform the opposite action. Thus, both E3 and DUBs must operate in a balanced manner to maintain hESC function or activate differentiation^26,27^. Remarkably, the levels of distinct E3 and DUB enzymes undergo dramatic changes during differentiation of mouse ESCs and cell reprogramming^6,9^. Recent studies have revealed that distinct proteins with DUB activity such as USP22^32,33^, USP44^34,35^ or the proteasome component Psmd14^6^ regulate the levels of core pluripotency transcription factors. Likewise, specific E3 enzymes modulate pluripotency and differentiation^29^. For instance, Huwe1 polyubiquitinates the pluripotency transcription factor N-myc, promoting its degradation by the proteasome and allowing for neural differentiation of mouse ESCs^36^.

To obtain further insights into the mechanisms by which increased proteasome activity sustain hESC identity, here we determine the interactome of highly-expressed E3 ligases in these cells (*i.e*., HERC2, UBE3A or RNF181). Moreover, we perform loss-of-function experiments of these E3s enzymes to examine their impact on hESC identity. Besides its role in the regulation of damaged proteins and endogenous substrates, increased proteasome activity could also be coupled to intrinsic characteristics of pluripotent stem cells. For instance, ESC and iPSCs exhibit up-regulated global translational rates^37^. To assess this hypothesis, we define the alterations induced by proteasome dysregulation using a shot label-free proteomic approach. Notably, we find changes in key biological processes of hESC identity such as RNA biogenesis, protein synthesis and telomere function, establishing the proteasome as a central regulator of hESCs.

## Results

### The levels of specific E3 enzymes decrease during differentiation

To obtain insights into the role of the UPS in hESC function, we examined changes in the expression of E3 ubiquitin ligases and DUBs during differentiation. For this purpose, we analysed available proteomics data comparing hESCs with their differentiated neuronal counterparts^9^ (Supplementary Data 1). We identified 31 DUBs in both hESCs and neurons. Among them, 10 were upregulated and 15 were downregulated during differentiation into neurons. We found that 44 out of 99 identified E3 enzymes increase during neuronal differentiation. In contrast, 35 E3 enzymes were decreased during differentiation into neurons (Supplementary Data 1 and Supplementary Table 1). To identify potential substrates of the UPS in hESCs, we further examined their up-regulated E3 ubiquitin ligases. Our approach was supported by the fact that three of these E3 enzymes were previously reported as regulators of ESC identity (*i.e*., HUWE1^36^, TRIM33^38^ and RNF40)^34^.

**Figure 1:**
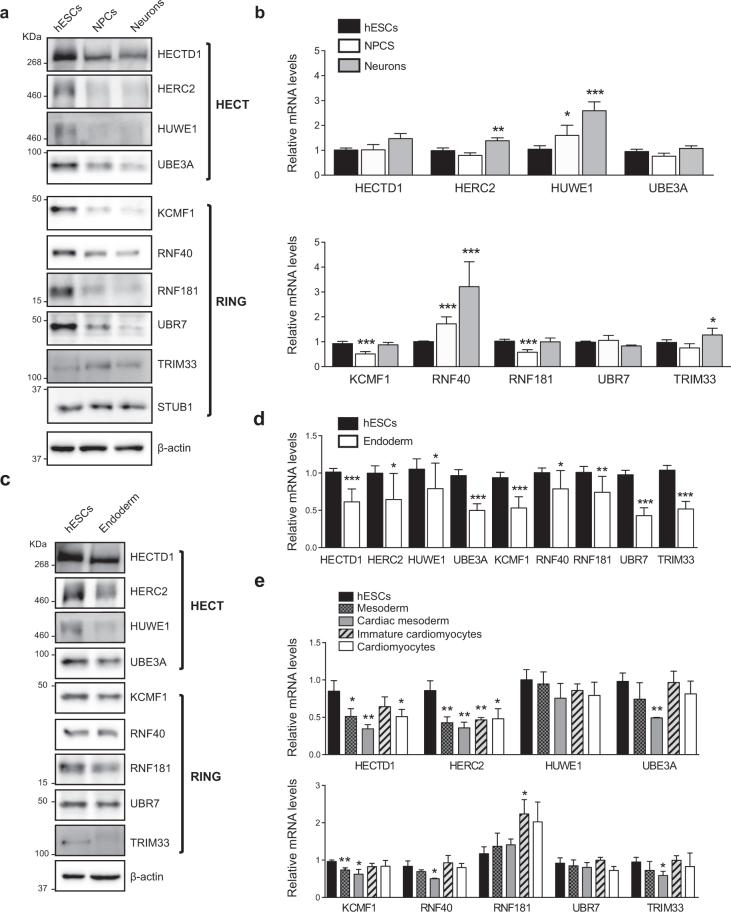
The levels of distinct E3 ubiquitin ligases decrease during differentiation. (**a**) Western blot analysis of H9 hESCs compared with their neural and neuronal counterparts (*i.e*., neural precursor cells (NPCs) and terminally differentiated neurons, respectively) with antibodies against distinct E3 ubiquitin ligases. ß-actin is the loading control. E3 enzymes are shown following their classification into HECT and RING-domain families. The images are representative of at least two independent experiments. (**b**) Quantitative PCR (qPCR) of E3 ubiquitin ligases mRNA levels. Graphs represent the mean ± s.e.m. (relative expression to H9 hESCs) of three independent cells. (**c**) Western blot analysis of E3 ubiquitin ligases comparing H9 hESCs with their differentiated endoderm precursors counterparts. ß-actin is the loading control. The images are representative of two independent experiments. All cropped blots were run under the same experimental conditions. Uncropped versions of western blots are presented in Supplementary Fig. 13. (**d**) qPCR analysis of E3 ubiquitin ligases transcript levels upon definitive endodermal differentiation of H9 hESCs. Graphs represent the mean ± s.e.m. (relative expression to H9 hESCs) of four independent cells. (**e**) mRNA levels of E3 enzymes in H1 hESCs and their differentiated mesoderm and cardiomyocyte counterparts. qPCR data represents the mean ± s.e.m. of three independent experiments. All the statistical comparisons were made by Student’s t-test for unpaired samples. P-value: *(P < 0.05), **(P < 0.01), ***(P < 0.001).

Among the 35 E3 enzymes significantly increased in hESCs, we found 6 ubiquitin ligases with HECT domain while the other E3 enzymes belonged to the RING-domain family^39^ (Supplementary Table 1). To validate these proteomics data, we performed western blot analysis of representative E3s of both families comparing hESCs with their neural progenitor cell (NPC) and neuronal counterparts. As a control, we also examined the levels of STUB1, an E3 enzyme which slightly increased during neuronal differentiation (Fig. 1a and Supplementary Data 1). At early neural stages, we already observed a downregulation in HECTD1, HERC2, HUWE1, UBE3A, KCMF1, RNF40, RNF181 and UBR7 levels (Fig. 1a). We corroborated the downregulation of distinct E3 ligases during neural differentiation in an independent hESC line (Supplementary Fig. 1). Moreover, these E3 enzymes were also decreased in terminally differentiated neurons validating our proteomics analysis (Fig. 1a and Supplementary Table 1). However, we could not confirm a decrease of TRIM33 levels during neurogenesis as we obtained inconsistent results among independent differentiation experiments (Fig. 1a and Supplementary Fig. 2).

The downregulation in the protein amount of the E3 ubiquitin ligases did not correlate with a reduction in their mRNA levels during neuronal differentiation (Fig. 1b), suggesting a post transcriptional or post-translational regulation of these enzymes. Since multiple E3 ligases are regulated by autoubiquitination rates^40^, decreased levels of E3s may also indicate their potential activation during differentiation. In hESCs, proteasome inhibition did not result in higher levels of the tested E3 enzymes (Supplementary Fig. 3a). Most importantly, proteasome inhibition did not up-regulate the levels of these E3 enzymes in neurons (Supplementary Fig. 3b). Thus, these data discard a faster turnover of our target E3 enzymes in differentiated cells induced by higher autoubiquination activity.

With the exception of RNF40, the protein levels of most of our target E3 enzymes were also decreased with differentiation into endoderm, indicating that this is not a specific phenomenon associated with the neural lineage (Fig. 1c). Among them, HECTD1, HERC2, HUWE1, UBE3A, RNF181 and TRIM33 were dramatically downregulated while KCMF1 and UBR7 were reduced to a lesser extent (Fig. 1c). In contrast to neuronal differentiation, we also observed a downregulation at the mRNA level (Fig. 1d). Moreover, the gene expression of many of these E3 enzymes (*e.g*., HECTD1, HERC2, KCMF1) was also downregulated when hESCs differentiated into mesodermal lineages (Fig. 1e). Overall, our results indicate that specific E3 enzymes such as HERC2, UBE3A and RNF181 are highly abundant in hESCs and decrease during differentiation.

### HECTD1 interacts with regulators of WNT signaling and glycolysis

**Figure 2:**
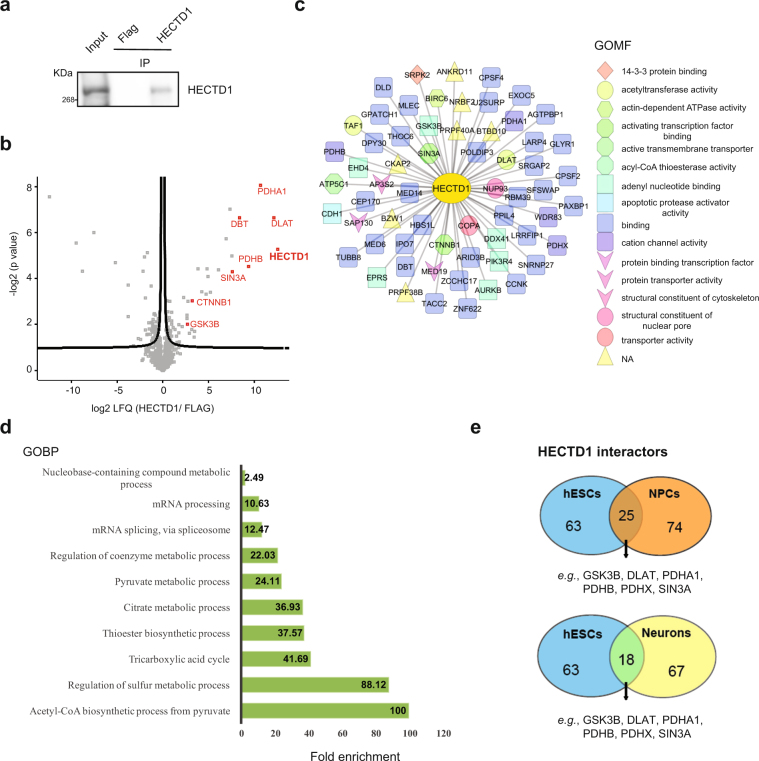
HECTD1 interacts with regulators of WNT signaling and glycolysis. (**a**) Co-immunoprecipitation (co-IP) with HECTD1 and FLAG antibodies in H9 hESCs followed by western blot against HECTD1. We loaded 13.3% of total input and 20% of total immunoprecipitated sample. The images are representative of three independent experiments. All cropped blots were run under the same experimental conditions. The original blots are included in Supplementary Fig. 13. (**b**) Volcano plot of the interactome of HECTD1 in H9 hESCs (n = 4). Graph represents the −log (p-value) of a two-tailed *t*-test plotted against the log2 ratio of protein label-free quantification (LFQ) values from co-IP experiments with HECTD1 antibody compared to control co-IP with FLAG antibody. Red colored dots beyond the curved lines indicate some of the most enriched interacting proteins after correction for multiple testing (False Discovery Rate (FDR) adjusted p-value (q-value) <0.2, s0 = 0.1). (**c**) Scheme indicating the Gene Ontology Molecular Function (GOMF) of HECTD1 interactors (Analysis tool: Cytoscape 3.6.0)^137^. (**d**) Bar graph representing the top GOBPs of HECTD1 interactome (P < 0.05) (Analysis tool: PANTHER^138^ and Gene Ontology Consortium). (**e**) Venn diagram represents total number and common significant interactors in hESCs, NPCs and neurons (FDR < 0.2 was considered significant, hESCs (n = 4), NPCs (n = 3) and neurons (n = 3).

To examine the role of up-regulated E3 enzymes in hESCs, we characterized their interactome. For this purpose, we performed co-immunoprecipitation experiments from hESCs followed by a single shot label-free proteomic approach. Our first target was HECTD1, a HECT E3 ubiquitin ligase involved in the negative regulation of β-catenin (CTNNB1). β-catenin is the main effector of WNT signaling^41^, one of the regulatory nodes of ESC pluripotency and neural differentiation^42,43^. We successfully immunoprecipitated significant levels of endogenous HECTD1 (Fig. 2a and Supplementary Fig. 4). Notably, interactome analysis indicated that HECTD1 binds β-catenin and GSK3B, a negative regulator of WNT signaling (Fig. 2b–c and Supplementary Data 2). We also found a strong interaction with subunits of the pyruvate dehydrogenase complex (PDC) (*i.e*., DLAT, PDHA1, PDHB, PDHX) (Fig. 2b–c). PDC catalyses the mitochondrial conversion of pyruvate into acetyl-CoA, which will be further metabolized through the tricarboxylic acid cycle and electron chain^44^. The metabolism of ESCs relies on glycolysis rather than mitochondrial respiration for energy production, a process achieved through an inactive PDC complex^45^. Thus, HECTD1 could participate in maintaining the glycolytic state of hESCs via modulation of PDC activity. In these lines, gene ontology biological process (GOBP) term analysis of the HECTD1 interactome indicated the strongest enrichment for proteins involved in the regulation of Acetyl-CoA biosynthetic process (Fig. 2d and Supplementary Data 2). Interestingly, SIN3A, a transcriptional regulatory protein required for both survival and pluripotency of ESCs^46,47^, also strongly interacted with HECTD1 (Fig. 2b–c), providing an additional putative link between HECTD1 and ESC function.

To determine whether HECTD1 binds these proteins specifically in hESCs, we performed co-immunoprecipitation experiments from NPCs and neurons. Among the 63 HECTD1 interactors identified in hESCs, 39.7% and 28.6% were also significant interactors in NPCs and neurons, respectively (Fig. 2e and Supplementary Data 3). These common interactors included GSK3B, PDC subunits (*i.e*., DLAT, PDHA1, PDHB, PDHX) and SIN3A in the three cell types tested. Thus, cell-type differences in HECTD1 abundance or activity could determine its regulatory impact on distinct pathways such as WNT signaling or Acetyl-CoA biosynthetic process. Our data also indicate a high percentage of specific interactions of HECTD1 depending on the cell type. Remarkably, 34 proteins interacted with HECTD1 in hESCs while we did not find a significant interaction in NPCs or neurons (Supplementary Data 3). Among these specific interactors, we found β-catenin as well as several regulators of cell cycle and proliferation (*e.g*., BIRC6, CCNK, CDH1, DDX41, PRPF40A, SRPK2, TAF1), providing a potential link of HECTD1 with the high division rates of these cells. We also identified a hESC-specific interaction of HECTD1 with AURKB, a regulator of telomerase activity in ESCs^48^. Moreover, HECTD1 specifically interacted in hESCs with proteins involved in gastrulation (*i.e*., DLD^49^) and embryonic cell differentiation (*e.g*., PAXBP1^50^, SRGAP2^51^ and THOC6)^52^. Thus, our data suggest that cell-specific interactions can also determine endogenous roles of up-regulated E3 enzymes in hESCs.

### Knockout of HERC2 alters the transcriptome of hESCs

**Figure 3:**
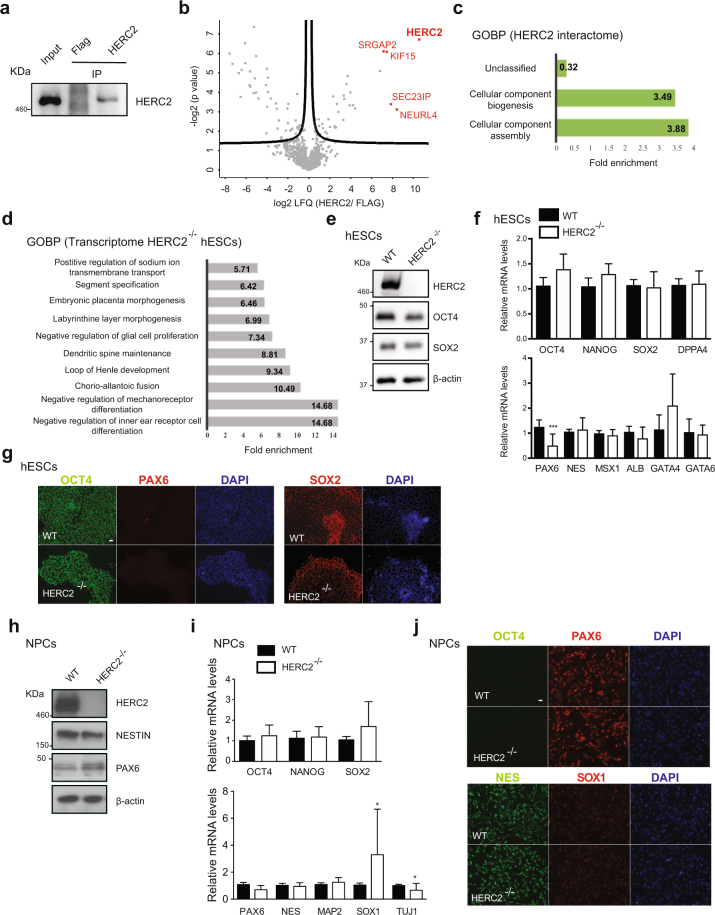
Knockout of HERC2 does not impair neural differentiation of hESCs. (**a**) co-IP with HERC2 and FLAG antibodies in H9 hESCs followed by western blot against HERC2. We loaded 13.3% of input and 20% of immunoprecipitated sample. The images are representative of three independent experiments. All cropped blots were run under the same experimental conditions. The original blots are included in Supplementary Fig. 13. (**b**) Volcano plot of the interactome of HERC2 treating H9 hESC samples with RNase A prior to immunoprecipitation (n = 4). Graph represents the –log (p-value) of a two-tailed *t*-test plotted against the log2 ratio of LFQ values from co-IP experiments with HERC2 antibody compared to control co-IP with FLAG antibody. Red colored dots indicate some of the most enriched interacting proteins after correction for multiple testing (FDR < 0.2, s0 = 0.1). (**c**) Bar graph representing the top GOBPs of HERC2 interactome (P < 0.05). (**d**) Bar graph representing the top GOBPs of the differentially expressed transcripts in *HERC2*^−/−^ H9 hESCs (P < 0.05). Transcripts showing a log_2_-fold change at a FDR < 0.05 were retained as significantly differentially expressed. (**e**) Western blot analysis with antibodies to HERC2, OCT4 and SOX2 comparing wild-type H9 (WT) with *HERC2*^−/−^ H9 hESCs. ß-actin is the loading control. The images are representative of two independent experiments. All cropped blots were run under the same experimental conditions. The original blots are included in Supplementary Fig. 13. (**f**) Real-time PCR analysis of pluripotency (upper panel) and germ-layer markers (lower panel) in *HERC2*^−/−^ H9 hESCs. Graphs (relative expression to wild-type H9 hESCs (WT)) represent the mean ± s.e.m. of four independent experiments. Statistical comparisons were made by Student’s t-test for unpaired samples. P-value: ***(P < 0.001). (**g**) Immunocytochemistry of H9 hESC with antibodies against OCT4 and SOX2 (markers for pluripotency) and PAX6 (neuroectodermal marker). DAPI staining was used as a marker of nuclei. Scale bar represents 20 μm. (**h**) Western blot analysis with antibodies to HERC2, NESTIN and PAX6 after 10 days of neural induction. ß-actin is the loading control. The images are representative of three independent experiments. (**i**) Real-time PCR analysis of pluripotency (upper panel) and neuroectodermal markers (lower panel) in H9 cells after 10 days of neural induction. Graphs (relative expression to WT) represent the mean ± s.e.m. of four independent experiments. Statistical comparisons were made by Student’s *t*-test for unpaired samples. P-value: *P < 0.05. (**j**) Immunocytochemistry after 10 days of neural differentiation of *HERC2*^−/−^ hESCs using antibodies against OCT4, PAX6, NES and SOX1. DAPI was used as a marker of nuclei. Scale bar represents 20 μm.

HERC2 is one of the most up-regulated E3 enzymes in hESCs when compared with their differentiated counterparts (Fig. 1a,c). HERC2 belongs to the HERC gene family, that contains a HECT domain and at least one RCC1-like domain (RLD). HERC2 interacts with chromatin via the RLD domain and participates in DNA repair, DNA replication as well as checkpoint control^53^. As such, HERC2 is located in a chromosomal region associated with neurodevelopmental disorders, including Prader-Willi and Angelman syndromes^54^. We immunoprecipitated endogenous HERC2 in hESCs (Fig. 3a and Supplementary Fig. 4) and identified 113 potential interactors (Supplementary Data 4). The three strongest interacting partners were SEC23IP, SRGAP2 and NEURL4 (Supplementary Fig. 5a), which are linked to development^51,55,56^. These proteins are already reported to interact with HERC2 in human cell lines, validating our data^57^. In differentiated cells, HERC2 has also been reported to interact with UBE3A, the primary E3 involved in Angelman syndrome^58^. Moreover, HERC2 has been recently associated with the kinase LRRK2, which plays a role in the familial Parkinson’s disease^59^. However, we did not observe these interactions in our proteomic data from hESCs, suggesting cell-type differences. On the other hand, the HERC2 interactome was strongly enriched for RNA-binding proteins (Supplementary Data 4). In addition, the most enriched GOBP pathways were related with RNA metabolism (Supplementary Fig. 5b and Supplementary Data 4). The high enrichment for pathways involved in RNA metabolism could indicate indirect interactions mediated by RNA and, therefore, ensuing from immunoprecipitation of RNA-protein complexes rather than direct protein-protein interactions^60^. To assess whether HERC2 directly interacted with these RBPs, we repeated the interactome analysis treating the samples with RNase A prior to immunoprecipitation with HERC2 antibody^60^. Whereas the interactions with SEC23IP, SRGAP2 and NEURL4 remained upon RNase treatment, the number of total significant interactors was reduced to 33 proteins (Fig. 3b and Supplementary Data 5). We found a significant interaction with centrosomal proteins (*i.e*., CENPF, CEP97) as well as RNA-binding proteins such as translation factors (*i.e*., EIF3A, EIF3C) and the helicase DDX20 (Supplementary Data 5). However, most of the interactions with RNA-binding proteins were lost upon RNase treatment. Moreover, GOBP analysis did not show an enrichment of RNA-related processes among these proteins (Fig. 3c and Supplementary Data 5). These data suggest that the previously identified RNA-binding proteins were immunoprecipitated due to secondary, RNA-mediated interactions.

With the high levels of HERC2 in hESCs and the role of one of its main interactors, SRGAP2, in neurogenesis and spine maturation^61^, we asked whether HERC2 regulates hESC function or differentiation. For this purpose, we generated a knockout hESC line by CRISPR/Cas9 genome editing (*HERC2*^−/−^) and performed transcriptome analysis. We found 1494 transcripts significantly changed in *HERC2*^−/−^ hESCs (Supplementary Data 6). Interestingly, GOBP analysis indicated strong enrichment for genes involved in the regulation of dendritic spine maintenance and regulation of glial cell proliferation (Fig. 3d and Supplementary Data 6). We also found an alteration in regulators of other developmental process, including nephron and placenta morphogenesis (Fig. 3d). Prompted by these findings, we examined whether these changes in the transcriptome impaired the levels of pluripotency markers. However, *HERC2*^−/−^ hESCs did not exhibit alterations in the levels of pluripotency markers when compared to control hESCs (Fig. 3e–g), indicating that that the sole deletion of HERC2 does not majorly impact on hESCs function. In addition, we did not find an increase in the expression of markers of the distinct germ layers (Fig. 3f), suggesting that loss of HERC2 does not induce differentiation. We only observe a decrease in the mRNA levels of PAX6, an early marker of neuroectodermal differentiation^62^ (Fig. 3f). At the pluripotent state, hESCs express marginal amounts of PAX6^63^ making these results difficult to interpret. Nevertheless, a decrease in PAX6 levels at the hESC stage could indicate a dysfunction in their ability to differentiate into NPCs. To assess this hypothesis, we induced neural differentiation. Besides a mild decrease in the mRNA levels of TUJ1, we did not find a significant downregulation in the induction of NPC markers at both protein and transcript levels (Fig. 3h,i). Most importantly, both control and *HERC2*^−/−^ cultures consisted mostly of PAX6-positive cells at the end of the neural induction treatment (Fig. 3j). Taken together, these results suggest that HERC2 has not a big impact in neural differentiation of hESCs.

### Highly abundant E3s interact with stem cell regulators in hESCs

**Figure 4:**
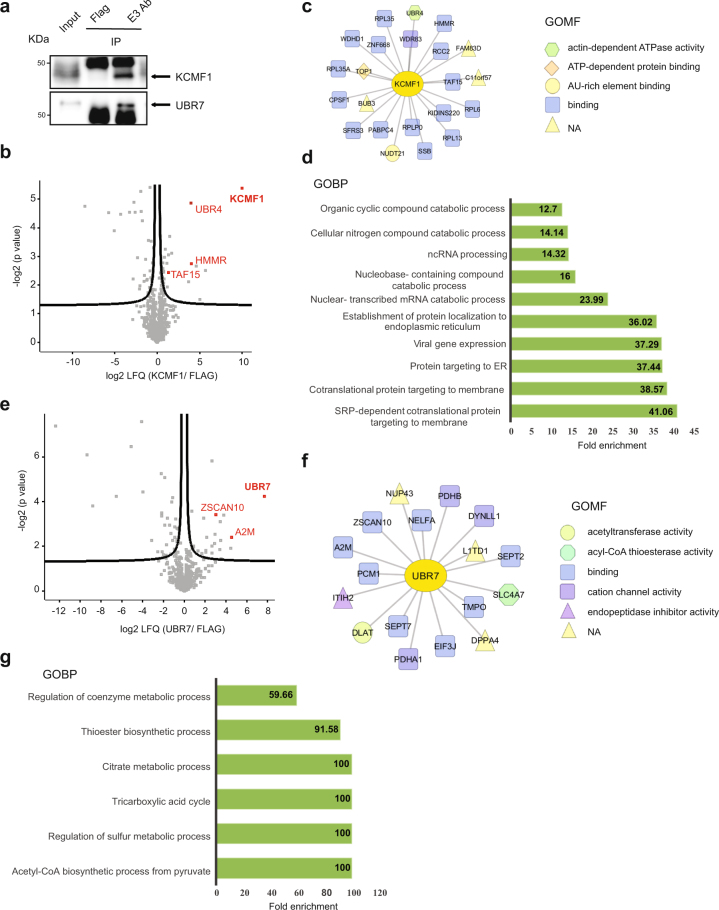
Analysis of the interactome of KCMF1 and UBR7 in H9 hESCs. (**a**) Co-IP with KCMF1, UBR7 and FLAG antibodies in H9 hESCs followed by western blot against the respective E3 ubiquitin ligases. We loaded 13.3% of input and 20% of immunoprecipitated sample. Arrows indicate the specific bands for KCMF1 and UBR7. The images are representative of three independent experiments. All cropped blots were run under the same experimental conditions. The original blots are included in Supplementary Fig. 13. (**b**) Volcano plot of the KCMF1 interactome (n = 4, FDR <0.2). Red colored dots indicate some of the most enriched interacting proteins after correction for multiple testing. (**c**) Scheme indicating the Gene Ontology Molecular Function (GOMF) of KCMF1 interactors (**d**) Bar graph representing the top GOBPs of KCMF1 interactome (P < 0.05). (**e**) Volcano plot of the UBR7 interactome (n = 4, FDR < 0.2). (**f**) Scheme indicating the GOMF of UBR7 interactors (**g**) Bar graph representing the top GOBPs of UBR7 interactome (P < 0.05).

We continued our interactome analysis of up-regulated E3 enzymes focusing on KCMF1, UBR7, UBE3A and RNF181 (Fig. 4a and Supplementary Fig. 4). We excluded HUWE1, TRIM33 and RNF40 as their role and targets have been previously defined in the context of hESC function^34,36,38^. KCMF1 is a poorly characterized RING finger-domain E3 enzyme^64^. In *Drosophila melanogaster*, KCMF1 orthologue regulates MAPK levels through its interaction with UBR4^65^, another E3 ubiquitin ligase. Moreover, the complex KCMF1-UBR4 promotes lysosomal degradation of the E2 ubiquitin protein RAD6^66^. We identified 23 significant KCMF1 interactors in hESCs, including UBR4 (Fig. 4b,c and Supplementary Data 7). One of the most enriched proteins was HMMR (Fig. 4b,c), an important regulator of stemness in murine ESCs through its role in signal transduction at microtubules^67^. TAF15, another significant KCMF1 interactor, has been previously reported as an epigenetic regulator of ESC pluripotency^68^. Among the most enriched GOBPs of KCMF1 interactors, we found processes involved in cotranslational protein targeting to the endoplasmic reticulum (Fig. 4d).

UBR7 belongs to the UBR-box family of E3 ligases. The UBR-box domain contains three zinc finger sites to bind proteins with modified N-terminal amino acid residues based on the N-end rule pathway^69^. In contrast to other members of the UBR-box family, little is known about UBR7. It was discovered in mammalian sperm, where it was shown to exhibit E3 ligase activity and suggested to be involved in spermiogenesis^70^. We identified 18 significant interactors of UBR7 in hESCs (Fig. 4e,f and Supplementary Data 8). The most enriched interactor was A2M, a regulator of cytokine availability in the local environment of hESCs and, thus, having a direct impact on pluripotency^71^ (Fig. 4e,f). Another strong UBR7 interactor was ZSCAN10, which is key for mouse ESC identity^72^. Similar to HECTD1, UBR7 interacted with distinct PDC subunits (*i.e*., DLAT, PDHA1 and PDHB) (Supplementary Data 8). Accordingly, GOBP analysis of UBR7 interactors indicated an enrichment in acetyl-CoA biosynthetic pathways (Fig. 4g).

**Figure 5:**
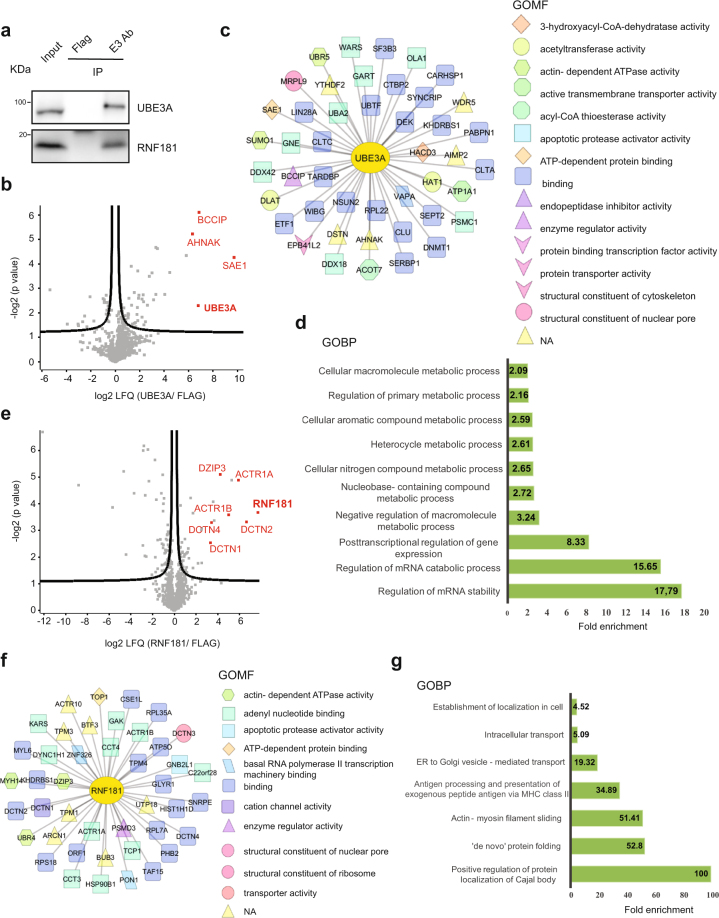
Highly abundant E3s interact with stem cell regulators in hESCs. (**a**) Co-IP with UBE3A, RNF181 and FLAG antibodies in H9 hESCs followed by western blot against the respective E3 ubiquitin ligases. We loaded 13.3% of input and 20% of immunoprecipitated sample. The images are representative of three independent experiments. All cropped blots were run under the same experimental conditions. The original blots are included in Supplementary Fig. 13. (**b**) Volcano plot of the UBE3A interactome (n = 3, FDR < 0.2). Red colored dots indicate some of the most enriched interacting proteins after correction for multiple testing. (**c**) Scheme indicating the Gene Ontology Molecular Function (GOMF) of UBE3A interactors (**d**) Bar graph representing the top GOBPs of UBE3A interactome (P < 0.05). (**e**) Volcano plot of the RNF181 interactome (n = 4, FDR < 0.2). (**f**) Scheme indicating the GOMF of RNF181 interactors (**g**) Bar graph representing the top GOBPs of RNF181 interactome (P < 0.05).

UBE3A, also known as E6-AP, belongs to the family of HECT E3 ubiquitin ligases and was initially discovered as a modulator of p53 degradation^73^. Genetic abnormalities in the maternally inherited UBE3A are responsible for Angelman syndrome^74,75^. Conversely, duplication or increased expression of *UBE3A* is linked to autism spectrum disorders^76^. UBE3A plays a role in dendritic arborization and synapse maturation^77–79^, as well as cell cycle progression^80^. Additionally, UBE3A participates in the clearance of several aggregated proteins^81,82^. Among the UBE3A interactors in hESCs, the most enriched protein was SAE1, an important regulator for reprogramming of human somatic cells^83^ (Fig. 5a–c and Supplementary Data 9). Another interactor of UBE3A was BCCIP, whose deficiency in mouse leads to impaired neural progenitor self-renewal and differentiation capabilities^84^. AHNAK, which was also significantly enriched in the UBE3A pull-down, is necessary for proper iPSC generation^85^. GOBP analysis of UBE3A interactors indicated enrichment for proteins involved in metabolic processes of cellular macromolecules, aromatic and nitrogen compounds as well as mRNAs (Fig. 5d and Supplementary 9). However, we cannot rule out that these interactions with RBPs involved in mRNA stability ensue from indirect RNA-mediated binding and further experiments will be required to asssess direct interaction (*e.g*., RNase A prior to immunoprecipitation).

The interactome of RNF181 was highly enriched for subunits of dynactin (specifically, DCTN2, ACTR1A, ACTR1B, DCTN4, DCTN1) (Fig. 5e,f and Supplementary Data 10), a macromolecular complex that regulates not only intracellular transport but also chromosome alignment and spindle organization during cell division^86^. Dynactin is necessary for proper asymmetric division of embryonic skin progenitors^87^ and thus may also play a role in the asymmetric divisions invoked by ESCs. Furthermore, RNF181 interacts with DZIP3, an E3 enzyme which regulates developmental genes in mouse ESCs by reorganizing 3D chromatin conformation^88^. Further experiments will be required to determine whether this interaction is direct or DNA-mediated. Besides the proteasome subunit PSMD3, RNF181 also interacted with distinct subunits of the TRiC/CCT chaperonin complex which is critical for hESC proliferation and differentiation^9^ (Fig. 5f). The TRiC/CCT complex facilitates the folding of ∼10% of the eukaryotic proteome and mediates protein localization to Cajal bodies, a sub-organelle found in the nucleus of highly proliferative cells, such as hESCs^89,90^. Accordingly, the most enriched GOBP of RNF181 interactors was the positive regulation of protein localization to Cajal bodies (Fig. 5g and Supplementary Data 10). Prompted by these findings, we examined whether RNF181 also interacts with these proteins in post-mitotic neuronal cells. Among the 46 RNF181 interactors found in hESCs, only 17% were also significant interactors in neurons (Supplementary Fig. 6 and Supplementary Data 11). Although RNF181 bound some of the dynactin subunits in neurons (*i.e*., DCTN2, ACTR1A, DCTN1), the interaction with DZIP3 as well as TRiC/CCT subunits was lost (Supplementary Fig. 6 and Supplementary Data 11). Taken together, the role of the interacting partners of up-regulated E3 ubiquitin ligases in hESCs indicated that these enzymes might be important for stem cell identity.

### Loss of distinct up-regulated E3 enzymes induces changes in the proteome of hESCs

**Figure 6:**
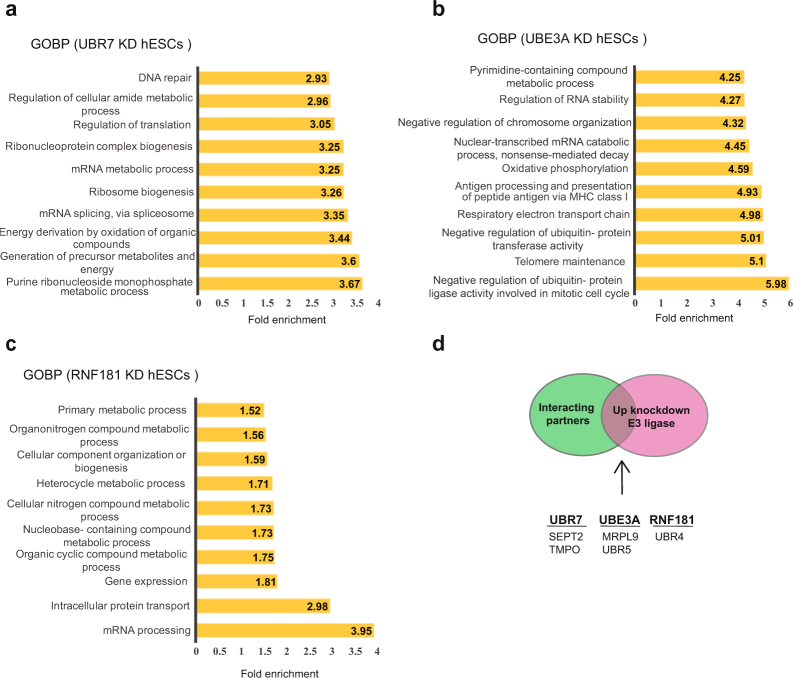
Loss of distinct up-regulated E3 enzymes induces changes in the hESC proteome. (**a**) Loss of UBR7 changed the levels of 506 proteins in H9 hESCs (FDR <0.2 was considered significant, n = 3). GOBP analysis (P < 0.05) revealed a strong enrichment for regulators of the purine metabolic process as well as generation of energy and metabolites. (**b**) Loss of UBE3A changed the levels of 661 proteins in H9 hESCs (FDR < 0.2 was considered significant, n = 3). Bar graph representing the top GOBPs changed upon UBE3A KD (P < 0.05). (**c**) Loss of RNF181 changed the levels of 202 proteins in H9 hESCs (FDR < 0.2, n = 3). Bar graph representing the top GOBPs changed upon RNF181 KD (P < 0.05). (**d**) Common proteins between interacting partners of E3 ubiquitin ligases and proteins up-regulated in hESCs with knockdown of the respective E3 enzymes.

To assess the role of up-regulated E3 ligases in hESC function, we generated stable knockdown (KD) lines for UBR7, UBE3A and RNF181 and analysed their proteome by quantitative proteomics (Supplementary Data 12). We identified the significantly changed proteins upon E3 knockdown and performed GOBP analysis (Supplementary Data 12). Loss of UBR7 changed the levels of 506 proteins, which were enriched for regulators of the generation of energy and metabolites as well as the purine metabolic process (Fig. 6a and Supplementary Data 12). These changes could have an impact on hESCs, which rely on highly active nucleotide synthesis rates for sustaining their rapid proliferation rates and *de novo* DNA and RNA production^91,92^. Among the 661 proteins changed upon UBE3A knockdown, GOBP analysis indicated the strongest enrichment for factors involved in the negative regulation of ubiquitin-ligase activity (Fig. 6b and Supplementary Data 12). This is consistent with previous studies, that showed that UBE3A interacts and ubiquitinates several proteasome subunits^93,94^ and regulates the activity of the proteasome in a ligase-dependent way^95,96^. Thus, UBE3A could be involved in the regulation of hESC identity through modulation of the proteasome, which is central for maintaining pluripotency^13^. Loss of RN181 changed the hESC proteome to a lesser extent (202 proteins) compared with UBR7 and UBE3A KD (Supplementary Data 12). Proteins dysregulated in RNF181 KD hESCs were involved mainly in mRNA processing-related pathways (Fig. 6c). Transcription hyperactivity has been proposed as a hallmark of pluripotent stem cells, as the transcription rates markedly decline during differentiation^97^. In these lines, mRNA-related proteins deregulated in RNF181 KD lines -such as SNRPD1, SRSF5 or HNRNPK- have been proven necessary for stemness in pluripotent cells^98–100^. If E3 interactors are activated for proteasomal degradation by the respective E3 enzyme, we would expect them to increase upon loss of the ubiquitin ligase. However, only a marginal number of the interacting partners were up-regulated upon E3 knockdown despite the numerous changes in the proteome induced by loss of E3 ligases (Fig. 6d).

**Figure 7:**
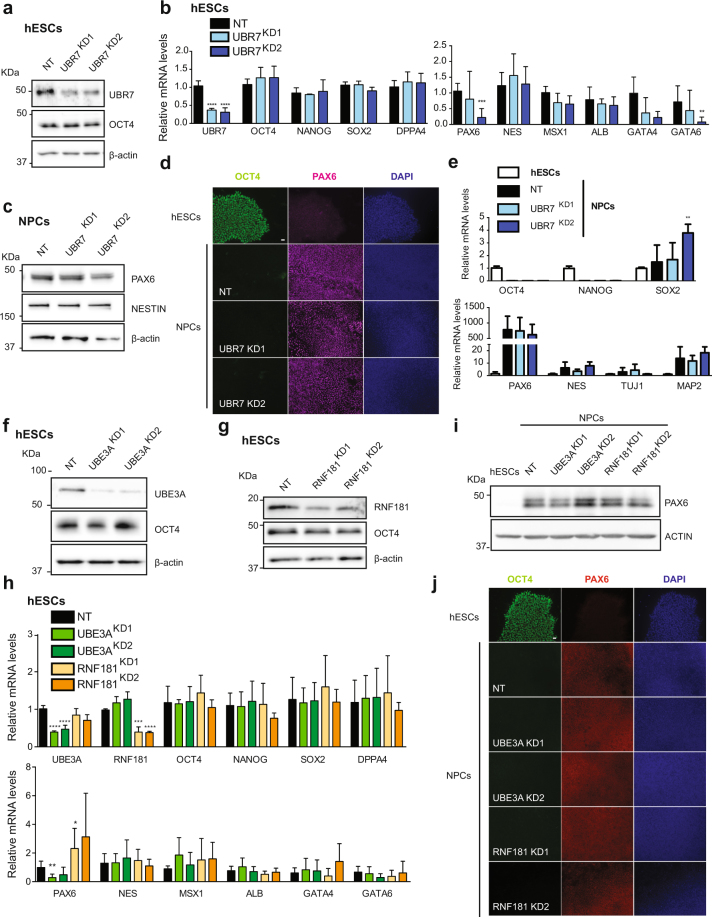
Loss of UBR7, UBE3A and RNF181 does not affect the expression of pluripotency markers and neural differentiation. (**a**) Western blot analysis with antibodies to UBR7 and OCT4 comparing non-targeting shRNA (NT) with UBR7 knockdown (KD) H9 hESCs. We used two independent shRNAs to UBR7 (KD1 and KD2, respectively). ß-actin is the loading control. The images are representative of two independent experiments. All cropped blots were run under the same experimental conditions. The original blots are included in Supplementary Fig. 13. (**b**) Real-time PCR analysis of pluripotency (left panel) and germ-layer markers (right panel) in UBR7 KD H9 hESCs. Graphs (relative expression to NT shRNA) represent the mean ± s.e.m. of four independent experiments. (**c**) Western blot analysis after 10 days of neural induction of UBR7 KDH9 hESCs. ß-actin is the loading control. The images are representative of two independent experiments. (**d**) Immunocytochemistry after 10 days of neural differentiation. OCT4, PAX6, and DAPI staining were used as markers of pluripotency, neuroectodermal differentiation, and nuclei, respectively. Scale bar represents 20 μm. (**e**) Real-time PCR analysis of pluripotency (upper panel) and neuroectodermal markers (lower panel) in UBR7 KD H9 hESCs after 10 days of neural differentiation. Graphs (relative expression to NT) represent the mean ± s.e.m. of three independent experiments. (**f**,**g**) Western blots of UBE3A and OCT4 (**f**) and RNF181 and OCT4 (**g**) protein levels. ß-actin is the loading control. The images are representative of two independent experiments. (**h**) Real-time PCR analysis of pluripotency (upper panel) and germ-layer markers (lower panel) in UBE3A and RNF181 KD H9 hESCs. Graphs (relative expression to NT shRNA) represent the mean ± s.e.m. of three independent experiments. (**i**) Western blot analysis after 10 days of neural induction of UBE3A and RNF181 KD H9 hESCs. ß-actin is the loading control. The images are representative of two independent experiments. (**j**) Immunocytochemistry after 10 days of neural differentiation. OCT4, PAX6, and DAPI staining were used as markers of pluripotency, neuroectodermal differentiation, and nuclei, respectively. Scale bar represents 20 μm. All the statistical comparisons were made by Student’s t-test for unpaired samples. P-value: *(P < 0.05), **P < 0.01, ***(P < 0.001), ****(P < 0.0001).

One step further was to examine whether loss of UBR7, UBE3A or RNF181 alters hESC identity and differentiation. Loss of UBR7 did not result in significant differences in pluripotency markers compared with control hESCs (Fig. 7a,b). Furthermore, the expression of markers of the distinct germ layers was not impaired (Fig. 7b). We induced neural differentiation of UBR7 KD hESCs and monitored the expression of PAX6. After 10 days of neural induction, PAX6 levels were increased at the same extent in control and UBR7 KD lines (Fig. 7c–e). We also analysed the levels of other neural and neuronal markers and found no differences at this stage (Fig. 7e). To assess whether UBR7 was required for proper differentiation into other germ lineages, we differentiated UBR7 KD hESCs into endoderm (Supplementary Fig. 7). The induction of distinct endoderm markers was similar between control and UBR7 KD lines, suggesting no major role of UBR7 in cell fate decisions towards the endodermal lineage (Supplementary Fig. 7).

We observed similar results in UBE3A and RNF181 KD hESCs, as pluripotency and differentiation markers remained unchanged after reducing the expression of these E3 enzymes (Fig. 7f–h). We also performed neural (Fig. 7i–j and Supplementary Fig. 8) and endodermal differentiation (Supplementary Fig. 9) from UBE3A and RNF181 KD hESCs and found no differences compared with control cells. Altogether, these results indicate that loss of UBR7, UBE3A or RNF181 alone does not alter the commitment of hESCs to a neuroectodermal or endodermal fate.

### Proteasome inhibition induces numerous changes in the proteome of hESCs

**Figure 8:**
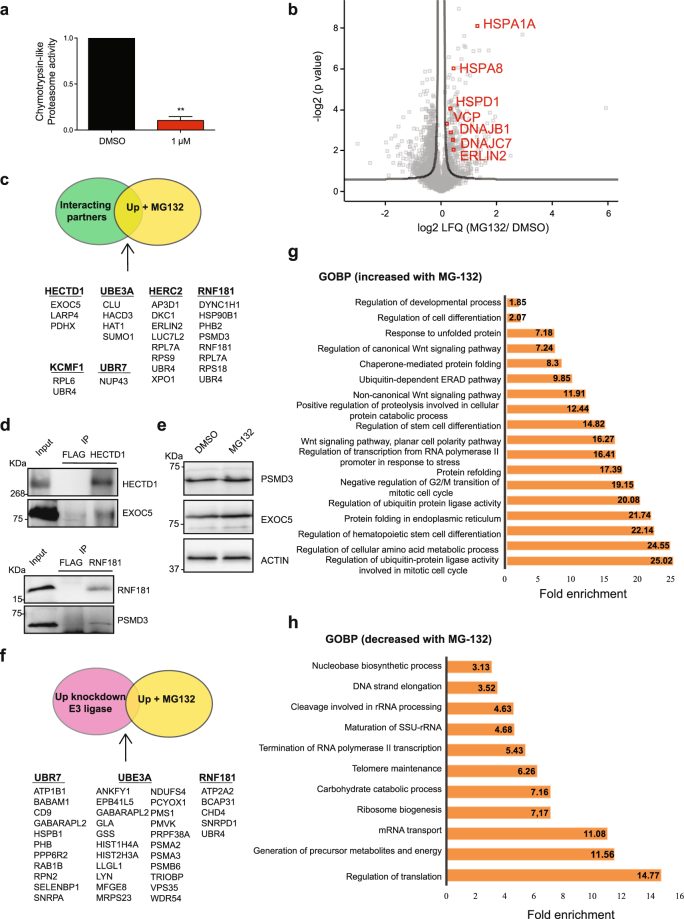
Proteasome inhibition induces profound changes in the proteome of ESCs. (**a**) Chymotrypsin-like proteasome activity in H9 hESCs treated with 1 µM MG-132 for 24 h (relative slope to H9 hESCs treated with DMSO). Graph represents the mean ± s.d. of two independent experiments. Statistical comparisons were made by Student’s t-test for unpaired samples. P-value: **(P < 0.01). (**b**) Volcano plot of LFQ intensities in MG-132-treated H9 hESCs as compared to control DMSO-treated hESCs. The significance of a two-tailed *t*-test is plotted against the log_2_ fold change of LFQs (FDR = 0.2, s0 = 1). Proteasome inhibitor: 1 µM MG-132 for 24 h. (**c**) Common proteins between interacting partners of the E3 ubiquitin ligases and proteins up-regulated in hESCs upon proteasome inhibition. (**d**) Co-IP with HECTD1, RNF181 and FLAG antibodies in H9 hESCs followed by western blot against EXOC5 and PSMD3. (**e**) Western blot analysis with antibodies to EXOC5 and PSMD3 of MG-132-treated H9 hESCs. ß-actin is the loading control. The images are representative of three independent experiments. All cropped blots were run under the same experimental conditions. The original blots are included in Supplementary Fig. 13. (**f**) Common up-regulated proteins between E3 knockdown hESCs and MG-132-treated hESCs. (**g**) Bar graph representing the top GOBPs of the up-regulated proteins upon proteasome inhibitor treatment (P < 0.05). (**h**) Bar graph representing the top GOBPs of the down-regulated proteins upon proteasome inhibitor treatment (P < 0.05).

To assess whether E3 enzymes potentially regulate proteasomal degradation of specific interactors, we induced acute proteasome dysfunction with the potent MG-132 proteasome inhibitor followed by a shot label-free proteomic approach (Fig. 8a,b). We identified 2735 proteins and hierarchical clustering indicated a clear separation of MG-132-treated hESCs compared with control hESCs based on global protein expression profiles (Supplementary Fig. 10). Quantitative analysis revealed that 899 proteins were significantly changed in MG-132-treated hESCs as compared to control hESCs (Fig. 8b and Supplementary Data 13). Among them, 552 proteins were down-regulated whereas 347 were up-regulated upon proteasome inhibition (Fig. 8b and Supplementary Data 13). Since the latter group could be putative proteasome targets, we integrated these data with the statistically significant E3 interactors. We identified several interactors of each E3 which were also up-regulated upon proteasome inhibitor (Fig. 8c). Among them, we confirmed by western blot the interactions of HECTD1 with EXOC5 and RNF181 with PSMD3 (Fig. 8d). Proteasome inhibition induced a slight increase in both EXOC5 and PSMD3 as we observed by proteomics and western blot (Fig. 8e, Supplementary Fig. 11 and Supplementary Data 13). Thus, a detailed analysis and validation of other E3 interacting partners could reveal novel proteasome targets in hESCs. However, it is also important to note the low number of up-regulated proteins commonly identified in E3 KD lines and proteasome inhibitor-treated hESCs (Fig. 8f). Among the 256 proteins up-regulated in UBR7 KD hESC, only 11 proteins were also increased upon proteasome inhibition (Fig. 8f). Loss of UBE3A resulted in 407 up-regulated proteins. Among them, 22 were also increased upon MG-132 treatment, including TRIOBP and MFGE8, both important for ESC function^101,102^ (Fig. 8f). Knockdown of RN181 only induced an increase in 4 proteins that were also up-regulated under proteasome inhibition (Fig. 8f). Thus, although a E3-based approach could lead to the discovery of potential proteasomal targets in hESCs, our results indicate that E3s are regulating multiple processes in a proteasome-independent manner that can complicate our analysis. Moreover, we cannot discard compensatory mechanisms or potential functional redundancies among different E3 enzymes^103–106^ as suggested by the lack of strong phenotypes in our loss-of-function experiments. In these lines, we have observed multiple common interactors between distinct E3 enzymes (Supplementary Fig. 12).

Another potential hypothesis is that enhanced proteasome activity of hESCs is not directly linked to the degradation of endogenous substrates. In this model, increased proteasome activity could be coupled with intrinsic characteristics of pluripotent stem cells. In support of this hypothesis, we observed a higher number of down-regulated proteins than increased proteins under proteasome inhibition (552 down-regulated *versus* 347 up-regulated). To gain insight into the effects of proteasome dysfunction in hESCs, we performed GOBP term analysis of these altered proteins. Analysis of up-regulated proteins indicated strong enrichment for genes involved in proteasomal-mediated ubiquitin degradation, including numerous proteasome subunits (Fig. 8g and Supplementary Data 13). Among them, we found an up-regulation of the proteasome activator PSMD11/RPN6^13^. Thus, these data suggest that hESCs trigger the expression of proteasome subunits as a compensatory mechanism to ameliorate the proteostasis decline induced by proteasome inhibition. In line with these results, we also found a marked up-regulation of proteins involved in chaperone-mediated protein folding and refolding (*e.g*., HSPA1A, DNAJC7, DNAJB1, HSPA8, HSPD1 and the stress-induced HSP90AA1), protein folding in the endoplasmic reticulum (ER) and ubiquitin-dependent ER-associated degradation (ERAD) (*e.g*., ERLIN2, VCP). Moreover, we found GOBP enrichment for regulators of stem cell differentiation, cell cycle and WNT signalling pathway (Fig. 8g and Supplementary Data 13). However, this enrichment was essentially due to increased levels of proteasome subunits, which are well-known regulators of the aforementioned biological processes.

On the other hand, GOBP term analysis of down-regulated proteins upon MG-132 treatment revealed a strong enrichment for factors involved in different pathways linked with hESC maintenance (Fig. 8h and Supplementary Data 13). For instance, proteasome dysfunction induced downregulation of activators of ribosome biogenesis and translation (Fig. 8h), a process that may compensate the decline in proteolytic ability. However, these changes could also trigger differentiation as ESCs rely on enhanced expression of ribosomal subunits and global translational rates to sustain their self-renewal and pluripotency^37,107^. Moreover, ESCs produce high levels of pre-rRNA^108^ and we found a down-regulation of proteins involved in maturation of rRNA (TSR1, HEATR1, EMG1) (Fig. 8h). In addition, proteasome dysfunction decreased the levels of distinct modulators of mRNA transport such as the nuclear pore complex protein NUP153, which is required for ESC pluripotency^109^.

Notably, proteasome inhibition also induced the down-regulation of factors involved in telomere maintenance and organization (*e.g*., RIF1, NOP10) (Fig. 8h), a key process for hESC maintenance^110,111^. Besides these changes, we found a decrease in the levels of multiple proteins involved in catabolic carbohydrate process (*e.g*., the glycogen phosphorylases PYGL/PYGB) as well as generation of precursor metabolites and energy (Fig. 8h). Remarkably, we observed a downregulation of hexokinase II (HK2), which is required for the high glycolytic rates of hESCs^92^. We also found a decrease in LIN28A (Supplementary Data 13), a core component of hESC identity that regulates the translation and stability of numerous mRNAs^112–115^, modulating key biological processes such as metabolism^116^. Thus, proteasome dysfunction impairs the protein levels of multiple factors involved in biological processes required for hESC function, suggesting a potential link between enhanced proteasome and other determinants of hESC identity such as metabolism, RNA biogenesis and telomere maintenance.

## Discussion

Pluripotent stem cells exhibit intrinsic high levels of proteasome activity^12^. Recent work demonstrates that this enhanced proteasome activity is essential for the striking ability of hESCs to maintain their proteostasis^9,12,13,19^. Besides this role in protein quality control, here we aimed to uncover additional molecular mechanisms by which proteasome activity regulates hESC function. First, we assessed whether increased proteasome is also required to regulate the concentration of endogenous targets in these cells by identifying highly-expressed E3 enzymes. Then, we characterized the interactome of the up-regulated E3 ligases and integrated these data with our proteomics experiments in E3-knockdown and proteasome inhibitor-treated hESCs. Unexpectedly, we identified a relatively low number of potential E3 substrates which were also increased upon proteasome inhibition. The difficulties in the identification of E3 substrates have been previously shown to be notoriously challenging^117,118^. First, protein ubiquitination is a highly dynamic process modulated by the coordinated action of E3 ligases and DUBs, which can remove the ubiquitin chains of E3 substrates. The balanced regulation of both competing processes could determine the fate of specific targets depending on the cellular status and environment. In this context, changes in the activity of specific DUBs could compensate the silencing of E3 enzymes. In a further level of complexity, DUBs can directly modulate the activity and turnover of E3 ligases^119^. Thus, a further understanding of the equilibrium between DUB and E3 activities in proteasomal degradation of pluripotency factors is of central importance. Secondly, the interaction between the E3 ligases and the substrates is rapid and weak. Hence, this transient interaction will make it especially challenging to isolate the complex E3-substrate^120^. Finally, we believe that significant degrees of redundancy and compensatory mechanisms among the E3 enzymes could be particularly relevant for our study. A substrate may be ubiquitinated by multiple E3s in the same or different lysines and even one single protein might be ubiquitinated by distinct enzymes under different conditions or even within single cellular compartments^121^. E3 substrates can be modified with a single ubiquitin or chains of ubiquitin at one or multiple sites. This variety in the ubiquitination generates a highly diverse topology of poly-ubiquitin chains, which not always targets the substrate towards degradation through the proteasome, but will have several different functions (*i.e*., intracellular signalling events)^122^. Thus, the interacting partners found in our proteomic analysis could not be substrates of the proteasome, but rather have their activity/function modified upon ubiquitination or simply be interacting proteins which might participate in an E3-containing protein complex. Nevertheless, our interactome analysis in hESCs may contribute to the understanding of the role of these E3 ubiquitin ligases.

In order to establish whether the E3 ubiquitin ligases have an impact on hESC identity, we silenced their expression and analysed stem cell pluripotency and differentiation capacities. However, we did not find a major effect on stem cell markers and their differentiation capacity upon loss of single E3 ubiquitin ligases. As mentioned above, that could be explained by potential functional redundancies between E3 ligases, as it has been previously shown for distinct E3s^105^. One example is the yeast transcriptional repressor alpha2, which can be independently marked for proteasomal degradation by the E3 enzymes Doa10 and Slx5/Slx8^106^. Moreover, the E3 ligases SAN1 and UBR1 can independently regulate the chaperone-dependant ubiquitination of misfolded proteins in yeast^103^. In mammalian cells, the tumour suppressor p53 can be degraded through the action of many E3 ligases, such as COP1, WWP1, E4F1 and PIRH2, which provides an additional layer of complexity in the regulation of the UPS^104^. These redundant mechanisms could ensure proper degradation of a particular substrate, even when one of the E3s might not be functional or overloaded by the number of targets to ubiquitinate. Furthermore, the different E3s might show subtle differences depending on their distinct subcellular localizations to enable intracellular-specific ubiquitination of the same substrate. Given the complex roles of E3s in maintaining cellular homeostasis and development, pluripotent stem cells likely rely on a tight coordination of redundant and compensatory mechanisms to maintain their function. Thus, simultaneous deletion of distinct E3 ligases might be necessary to induce a strong demise in their pluripotency. In addition, we cannot discard that the lack of phenotype of E3-depleted hESCs ensues from the activation of other proteostatic pathways that counterbalance UPS disturbances. For instance, it has been demonstrated that inhibition of proteasome induces autophagy as a compensatory response, in a p62-mediated manner^123,124^.

In parallel to this approach, we also performed quantitative proteomic analysis of hESCs treated with proteasome inhibitor. By integrating these data with our interactome and loss of function experiments for distinct E3 ligases, we identified potential targets of the proteasome in hESCs. Most importantly, global proteasome inhibition experiments showed that besides its key role in maintaining proteostasis in hESCs, the proteasome is also coupled to other determinant biological processes of hESC identity, such as their intrinsic metabolism, RNA biogenesis and telomere maintenance. Overall, here we provide a comprehensive characterization of the UPS system in hESCs that could have important implications for a further understanding of these cells.

## Experimental Procedures

### hESCs culture and differentiation

The human H9 (WA09) and H1 (WA01) lines were obtained from WiCell Research Institute. H9 and H1 were maintained on Geltrex (ThermoFisher Scientific) using mTeSR1 (Stem Cell Technologies). Undifferentiated hESCs colonies were passaged using a solution of dispase (2 mg ml^−1^) and scraping the colonies with a glass pipette. All the cell lines used in this study were tested for mycoplasma contamination at least once every three weeks and no mycoplasma contamination was detected. All research involving hESCs lines was performed with approval of the German Federal competent authority (Robert Koch Institute) and in accordance with the relevant guidelines and regulations.

Neural differentiation of H9 cell line was performed following the monolayer culture method as described previously^125^ with STEMdiff Neural Induction Medium (Stem Cell Technologies) on polyornithine (15 μg ml^−1^)/laminin (10 μg ml^−1^)-coated plates. Undifferentiated hESCs were rinsed once with PBS and then we added 1 ml Gentle Dissociation Reagent (Stem Cell Technologies) for 5 min. After incubation at 5 min, we gently collected hESCs and 2 ml of Dulbecco’s Modified Eagle Medium (DMEM)-F12 + 10 μM ROCK inhibitor (Abcam). Afterward, we centrifuged cells at 300 g for 10 min. Cells were resuspended on STEMdiff Neural Induction Medium + 10 μM ROCK inhibitor and plated on polyornithine (15 μg ml^−1^)/laminin (10 μg ml^−1^)-coated plates. For neuronal differentiation, NPC at passage 4 were dissociated with Accutase (Stem Cell Technologies) and plated into neuronal differentiation medium (DMEM/ F12, N2, B27 (ThermoFisher Scientific), 1 mg ml^−1^ laminin (ThermoFisher Scientific), 20 ng ml^−1^ brain-derived neurotrophic factor (BDNF) (Peprotech), 20 ng ml^−1^ glial cell-derived neurotrophic factor (GDNF) (Peprotech), 1 mM dibutyryl-cyclic AMP (Sigma) and 200 nM ascorbic acid (Sigma)) onto polyornithine/laminin-coated plates. Cells were differentiated for 1 month, with weekly feeding of neuronal differentiation medium. Endoderm differentiation of H9 hESCs was performed using STEMdiff Definitive Endoderm Kit (Stem Cell Technologies).

Cardiomyocyte differentiation of H1 hESCs was performed as described in ref.^126^. Confluent H1 hESCs were dissociated into single cells with Accutase at 37 °C for 10 min followed by inactivation using two volumes of F12/DMEM. Cells were counted and 230,000 cells cm^2^ where plated in ITS medium (Corning), containing 1.25 mM CHIR 99021 (AxonMedchem) and 1.25 ng ml^−1^ BMP4 (R&D), and seeded on Matrigel-coated 24-well plates. After 24 h, medium was changed to TS (transferrin/selenium) medium. After 48 h, medium was changed to TS medium supplemented with 10 mM canonical Wnt-Inhibitor IWP-2 (Santa Cruz) for 48 h. Then, medium was changed to fresh TS until beating cells were observed at days 8–10. Finally, medium was changed to Knockout DMEM (ThermoFisher Scientific) supplemented with 2% FCS, L-Glutamine and Penicillin/Streptomycin until cells were used for downstream analysis.

### Lentiviral infection of H9 hESCs

Lentivirus (LV)-non-targeting shRNA control, LV-UBR7 shRNA #1 (TRCN0000037025), LV-UBR7 shRNA #2 (TRCN0000037026), LV-UBE3A shRNA #1 (TRCN0000419838), LV-UBE3A shRNA #2 (TRCN0000003368), LV-RNF181 shRNA #1 (TRCN0000364405) and LV-RNF181 shRNA #2 (TRCN0000022389) in pLKO.1-puro vector were obtained from Mission shRNA (Sigma). For generation of stable hESCs transfected lines, H9 hESCs growing on Geltrex were incubated with 10 μM ROCK inhibitor for one hour and then individualized using Accutase. Fifty thousand cells were infected in suspension with 25 μL of concentrated virus in the presence of 10 μM ROCK inhibitor. Cell suspension was centrifuged to remove the virus and plated back on a feeder layer of mitotically inactivated MEF in CDF12 media containing ROCK inhibitor (DMEM/F12 plus 20% Knock-out Serum Replacement, β-mercaptoethanol (0.1%), 0.1 mM non-essential-aminoacids, Glutamax (1%), and 10 ng/mL bFGF). After few days in culture, cells were selected for lentiviral integration with 2 µg ml^−1^ puromycin (ThermoFisher Scientific) and remaining colonies were manually passaged onto fresh MEFs to establish new hESC lines.

### Generation of *HERC2*^−/−^ hESCs by CRISPR/Cas9 system and colony analysis

*HERC2* genomic sequence was obtained from ENSEMBL Genome browser. Two guide sequences (Guide A, targeting the intron 4–5, F: CCTCAGTTTCTTCATCCATAAAAC, and R: CAGCACTGCTTGACAGTGCTGGG; Guide B, targeting the fifth exon, F: CCGTCCAGTCAGCCACCACCACC, and R: CAGCCCTGCGACTCAAGCAGAGG), were generated using the Zinc Finger Consortium online resource (http://zifit.partners.org/ZiFiT/). Guide-carrying plasmid were designed as previously described in refs^127,128^ using Cas9-puromycine selection plasmid (pX335-U6-Chimeric_BB-CBh-hSpCas9n(D10A) was a gift from Feng Zhang (Addgene plasmid # 42335)). H9 hESCs were plated on MEF-containing plates and transfected with 1 μg of each of the guide-carrying plasmids using FuGene HD (Promega). 24 h after the transfection, 0.5 μg ml^−1^ puromycine selection was performed for 24 h followed by maintenance of hESCs with CDF12 media. Single cell split was performed prior to colony pick for genotyping. Genomic DNA isolation was done using QuickExtract (Epicentre). PCR using primer pairs surrounding HERC2 fifth exon was performed (Forward: TAGAGAGAGGCAGTGTGCCA, and Reverse: TGGCTGGCTTCCACAGTTAC) to identify *HERC2*^−/−^ hESCs.

### Immunoprecipitation of HERC2, HECTD1, RNF181, KCMF1 and UBR7 for interaction analysis

hESCs were lysed in RIPA buffer (50 mM Tris-HCl (pH 6.7), 150 mM NaCl, 1% Triton x-100, 1% sodium deoxycholate, 1 mM EDTA, 1 mM PMSF) and supplemented with protease inhibitor (Roche). Lysates were homogenized through syringe needle (27 G) and centrifuged at 13,000 *g* for 15 min at 4 °C. For RNase A-treated samples, the supernatant was incubated with 125 µl ml^−1^ RNAse A (ThermoFischer) for one hour on ice. Otherwise, incubation with the antibody was performed. 330 µg of protein-containing samples were incubated for 30 minutes with HERC2 antibody (Abcam, # ab85832, 4 µg) or UBR7 antibody (Thermo Scientific, # PA5-31559, 4 µg) or KCMF1 antibody (Abcam, # ab80287, 4 µg) or RNF181 antibody (Thermo Scientific, PA5-31008, 4 µg) or HECTD1 antibody (Abcam, #ab101992, 4 µg) on ice. As a control, the same amount of protein was incubated with anti-FLAG antibody (SIGMA, F7425, 4 µg). Subsequently samples were incubated with 100 µl of µMACS Micro Beads for 1 h at 4 °C with overhead shaking. After incubation, samples were loaded to precleared µMACS column (# 130-042-701). Beads were washed three times with 50 Mm Tris (pH 7.5), 150 mM NaCl, 5% glycerol, 0.05% Triton and then five times with 50 Mm Tris (pH 7.5) and 150 mMNaCl. For elution of the proteins for further Western Blot analysis, the beads were incubated with 50 µl of pre-heated (95 °C) 1X Laemmli Buffer for 5 minutes and collected into eppendorf tubes. The samples were boiled for 5 minutes at 95 °C and loaded in a SDS-PAGE gel. In Supplementary Fig. 4, we also show flow-through controls, that correspond to the supernatant that remains after centrifuging the tubes containing the antibody as well as the beads. For proteomics analysis, after the washes we performed partial digestion on the beads using 25 µl of 2 M Urea in 25 mM Tris, 7.5 Mm ammonium bicarbonate, 1 mM DTT and 5 ng/ml trypsin, pH = 8.0 for 30 minutes at room temperature. The samples were then eluted twice with 50 µl of 2 M Urea, 25 mM Tris, 7.5 mM ammonium bicarbonate, 5 mM Iodacetamide and left for fully digestion overnight at room temperature in the dark. For stopping the digestion, 5 µl of 100% formic acid was added to samples. Peptides were cleaned up using stage tip extraction^129^.

### Immunoprecipitation of UBE3A for interaction analysis

hESCs were lysed in RIPA buffer (50 mM Tris-HCl (pH 7.4), 150 mM NaCl, 1% Triton x-100, 1% sodium deoxycholate, 0.1% SDS, 1 mM EDTA, 1 mM PMSF) supplemented with protease inhibitor (Roche). Lysates were homogenized through syringe needle (27 G) and centrifuged at 13,000 *g* for 15 min at 4 °C. After preclearing the supernatant with Protein A agarose beads (Pierce), the samples were incubated overnight with UBE3A antibody (Cell Signaling, # 7526 1:50) on the overhead shaker at 4 °C. As a control, the same amount of protein was incubated with anti-FLAG antibody (SIGMA, F7425, 1:50) in parallel. Subsequently, samples were incubated with 30 µl of Protein A beads for 1 h at room temperature. After this incubation, samples were centrifuged 5 minutes at 5,000 g at room temperature and the pellet was washed three times with the RIPA buffer. For elution of the proteins for further western blot analysis, the pellet was incubated with pre-heated (95 °C) 2X Laemmli Buffer, boiled for 5 minutes and centrifuged 5 minutes at maximum speed. The supernatant was taken and loaded in a SDS-PAGE gel. For further proteomics analysis, after the washing steps, partial digestion of the proteins was performed on the agarose beads using 25 µl of 2 M Urea in 25 mM Tris, 7.5 mM ammonium bicarbonate, 1 mM DTT and 5 ng/ml trypsin, pH = 8.0 for 30 minutes at room temperature. Then, the samples were eluted 2 times with 50 µl of 2 M Urea, 25 mM Tris, 7.5 mM AmBic 5 mM Iodacetamide and were fully digested overnight at room temperature in the dark. For stopping the digestion, 5 µl of 100% formic acid was added to the samples. Peptides were cleaned up using stage tip extraction^129^.

### Sample preparation for quantitative proteomics and analysis

For analysis of MG132 inhibited cells and hESCs, NPC and neurons we performed tandem mass tag (TMT) proteomics. Cells were collected in 8 M urea buffer, 50 mM ammonium bicarbonate, homogenized with a syringe and cleared using centrifugation (16,000 g, 20 min). Supernatants were reduced by addition of DTT to a final concentration of 5 mM and incubated at room temperature for 30 minutes. Iodoacetamide was added to the samples to a final concentration of 5 mM to alkylate the cysteines and incubated in the dark for 45 minutes. Lys-C (Wako, Richmond, VA) was added for digestion at a ratio 1:100 enzyme/substrate and incubated for three hours at room temperatures. Then, lysates were diluted to 2 M Urea with 50 mM ammonium bicarbonate, after which 10 ng μl^−1^ Trypsin was added and incubated overnight at room temperature. Trypsin digestion was stopped by addition of formic acid and peptides were cleaned up using stage tip extraction^129^. The liquid chromatography tandem mass spectrometry (LC-MS/MS) equipment consisted out of an EASY nLC 1000 coupled to the quadrupole based QExactive instrument (Thermo Scientific) via a nano-spray electroionization source. Peptides were separated on an in-house packed 50 cm column (1.9 µm C18 beads, Dr. Maisch) using a binary buffer system: A) 0.1% formic acid and B) 0.1% formic acid in ACN. The content of buffer B was raised from 7% to 23% within 120 min and followed by an increase to 45% within 10 min. Then, within 5 min buffer B fraction was raised to 80% and held for further 5 min after which it was decreased to 5% within 2 min and held there for further 3 min before the next sample was loaded on the column. Eluting peptides were ionized by an applied voltage of 2.2 kV. The capillary temperature was 275 °C and the S-lens RF level was set to 60. MS1 spectra were acquired using a resolution of 70,000 (at 200 m/z), an Automatic Gain Control (AGC) target of 3e6 and a maximum injection time of 20 ms in a scan range of 300–1750 Th. In a data dependent mode, the 10 most intense peaks were selected for isolation and fragmentation in the HCD cell using a normalized collision energy of 25 at an isolation window of 2.1 Th. Dynamic exclusion was enabled and set to 20 s. The MS/MS scan properties were: 17.500 resolution at 200 m/z, an AGC target of 5e5 and a maximum injection time of 60 ms. All label-free proteomics data sets were analysed with the MaxQuant software^130^ (release 1.5.3.8). We employed the LFQ mode^131^ and used MaxQuant default settings for protein identification and LFQ quantification. All downstream analyses were carried out on LFQ values with Perseus (v. 1.5.2.4)^132^.

### Quantitative proteomics analysis of E3 enzymes

For the characterization of protein expression differences in E3 enzymes comparing H9 hESCs with their neuronal counterparts, we analysed the quantitative proteomics data published in ref.^9^. Statistical comparisons were made by Student’s t-test. Discovery Rate (FDR) adjusted p-value (q-value) was calculated using the Benjamini–Hochberg procedure. All the proteins at a FDR level below 0.2 were retained as significantly differentially expressed (Supplementary Data 1). Then, we intersected the human E3 network annotated in^133^ with this proteomics dataset (Supplementary Table 1).

### RNA sequencing

Total RNA was extracted using RNAbee (Tel-Test Inc.). Libraries were prepared using the TruSeq Stranded mRNA Library Prep Kit. Library preparation started with 1 µg total RNA. After selection (using poly-T oligo-attached magnetic beads), mRNA was purified and fragmented using divalent cations under elevated temperature. The RNA fragments underwent reverse transcription using random primers followed by second strand cDNA synthesis with DNA Polymerase I and RNase H. After end repair and A-tailing, indexing adapters were ligated. The products were then purified and amplified (20 µl template, 14 PCR cycles) to create the final cDNA libraries. After library validation and quantification (Agilent 2100 Bioanalyzer), equimolar amounts of library were pooled. The pool was quantified by using the Peqlab KAPA Library Quantification Kit and the Applied Biosystems 7900HT Sequence Detection System. The pool was sequenced on an Illumina HiSeq. 4000 sequencer with a paired- end (2 × 75 bp) protocol. We used the human genome sequence and annotation (EnsEMBL 79) together with the splice-aware STAR read aligner^134^ (release 2.5.1b) to map and assemble our reference transcriptome. Subsequent transcriptome analyses on differential gene and transcript abundance were carried out with the cufflinks package^135^.

### Western Blot

Cells were scraped from tissue culture plates and lysed in non-denaturing lysis buffer (10 mM Tris-HCL, pH 7.4, 10 mM EDTA, 50 mM NaCl, 50 mM NaF, 1% Triton X-100, 0.1%SDS, supplemented with 2 mM sodium orthovanadate, 1 mM PMSF and protease inhibitor (Roche)) and homogenized through a syringe needle (27 G). Cell lysates were centrifuged at 15,000 *g* for 15 min at 4 °C and protein concentration was measured from the supernatant using BCA protein assay (Thermo Scientific). Approximately 30 μg of total protein was separated by SDS–PAGE, transferred to PVDF membranes (Millipore) and subjected to immunoblot. Western blot analysis was performed with anti-HERC2 antibody (Abcam, # ab85832 1:2000), anti- UBR7 antibody (Thermo Scientific, # PA5-31559 1:2000), anti- KCMF1 antibody (Abcam, # ab80287, 1:3000), anti-RNF181 antibody (Thermo Scientific, PA5-31008, 1:2000), anti- HECTD1 antibody (Abcam, #ab101992, 1:1000), anti-UBE3A antibody (Cell Signaling, # 7526 1:1000), anti-RNF40 (Cell Signaling, #12187, 1:1000), anti-HUWE1 (Cell Signaling, #5695, 1:1000) anti-TRIM33 (Cell Signaling, #13387, 1:1000), anti-STUB1 (Cell Signaling, #2080, 1:1000), anti-OCT4 (Abcam, #ab19857, 1:5000), anti-SOX2 (Abcam, #97959, 1:1000), anti-PAX6 (Stem Cell Technologies, #60094, 1:1000), anti-NESTIN (Stem Cell Technologies, # 60091, 1:1000), anti-PSMD3 (Proteintech, # 12054-1-AP, 1:1000), anti-EXOC5 (Proteintech, # 17593-1-AP, 1:1000) and anti-ß-actin (Abcam, #8226, 1:10000). Uncropped versions of western blots are presented in Supplementary Fig. 13.

### Immunocytochemistry

Cells were fixed with paraformaldehyde (4% in PBS) for 20 min, followed by permeabilization (0.2% Triton X-100 in PBS for 10 min) and blocking (3% BSA in 0.2% Triton X-100 in PBS for 10 min). Human cells were incubated in primary antibody for 1,5 h at room temperature with anti-OCT4 (Stem Cell Technology, #60093, 1:200), anti-PAX6 (Stem Cell Technology, #60094, 1:300) or anti-SOX1 (Stem Cell Technology, #60095, 1:100). Then, cells were washed with PBS and incubated with secondary antibody (Alexa Fluor 488 goat anti-mouse (Thermo Fisher Scientific, A-11029), Alexa Fluor 568 F(ab’)2 Fragment of Goat Anti-Rabbit IgG (H + L) (Life Technologies, #A21069) and DAPI (Life Technologies, #1306) for 45 minutes at room temperature. PBS and distilled water wash were followed before the cover slips were mounted on Mowiol (Sigma, #324590).

### RNA isolation and quantitative RT–PCR

Total RNA was isolated using RNAbee (Tel-Test Inc.). cDNA was generated from 1 μg of RNA using qScript Flex cDNA synthesis kit (Quantabio). SybrGreen real-time qPCR experiments were performed with a 1:20 dilution of cDNA using a CFC384 Real-Time System (Bio-Rad) following the manufacturer’s instructions. Data were analysed with the comparative 2ΔΔ*C*_t_ method using the geometric mean of *ACTB* and *GAPDH* as housekeeping genes. See Supplementary Information for details about the primers used for this assay.

### 26S proteasome fluorogenic peptidase assays

The *in vitro* assay of 26 S proteasome activities was performed as previously described^136^. Cells were collected in proteasome activity assay buffer (50 mM Tris-HCl, pH7.5, 250 mM sucrose, 5 mM MgCl2, 0.5 mM EDTA, 2 mM ATP and 1 mM DTT) and lysed by passing ten times through syringe needle (27 G). Then, lysate was centrifuged at 10,000 g for 10 min at 4 °C. 25 μg of total protein of cell lysates were transferred to a 96-well microtiter plate (BD Falcon) and incubated with the fluorogenic substrate Z-Gly-Gly-Leu-AMC (Enzo Lifescience). Fluorescence (380 nm excitation, 460 nm emission) was monitored on a microplate fluorometer (EnSpire, Perkin Elmer) every 5 min for 2 h at 37 °C.

### Data availability

All the mass spectrometry proteomics data as well as the transcriptomic data are available in the Supplementary Information. All the other data are also available from the corresponding author upon request.

## Electronic supplementary material

Supplementary Information

Dataset 1

Dataset 2

Dataset 3

Dataset 4

Dataset 5

Dataset 6

Dataset 7

Dataset 8

Dataset 9

Dataset 10

Dataset 11

Dataset 12

Dataset 13
