# Supplementary material for: Insights into the ubiquitin-proteasome system of human embryonic stem cells
Source: Sci Rep. 2018 Mar 6;8:4092. doi: 10.1038/s41598-018-22384-9 (PMC5840266; doi:10.1038/s41598-018-22384-9)
Supplement: Supplementary file 1 — Supplementary Information [file 41598_2018_22384_MOESM1_ESM.pdf]

Supplementary Information

Insights into the ubiquitin-proteasome system of human embryonic stem cells

Isabel Saez[1#], Seda Koyuncu[1#], Ricardo Gutierrez-Garcia[1], Christoph Dieterich[2] and David Vilchez[1]

[1] Institute for Genetics and Cologne Excellence Cluster for Cellular Stress Responses in Aging-Associated Diseases (CECAD), University of Cologne, Joseph-Stelzmann-Strasse 26, 50931 Cologne, Germany.

[2] Section of Bioinformatics and Systems Cardiology, Department of Internal Medicine III and Klaus Tschira Institute for Computational Cardiology, Neuenheimer Feld 669, University Hospital, 69120 Heidelberg, Germany.

[#] These authors contributed equally to this work.

Supplementary Figure 1

H1 hESCs

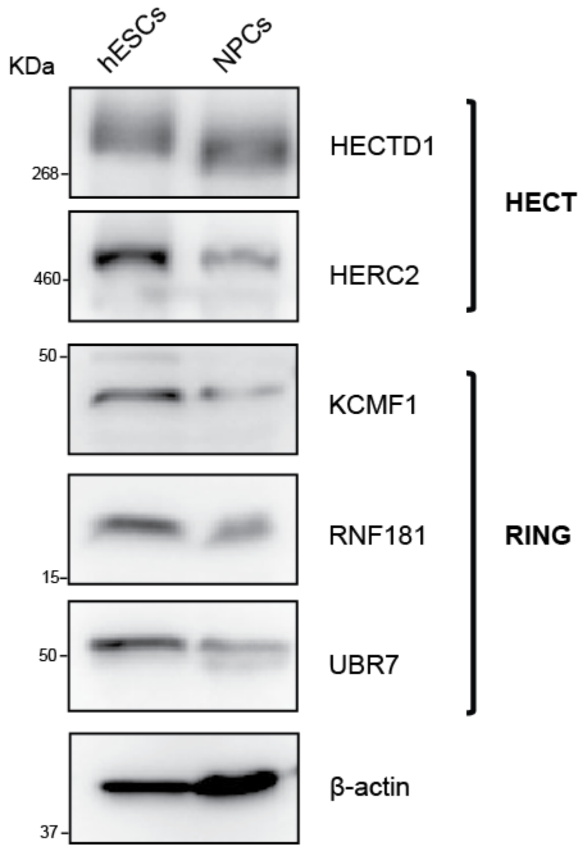

Supplementary Figure 1. Analysis of E3 protein levels in H1 hESCs. Western blot with antibodies against HECTD1, HERC2, KCMF1, RNF181 and UBR7 comparing H1 hESCs with their neural (NPC) counterparts. ß-actin is the loading control.

Supplementary Figure 2

H9 hESCs

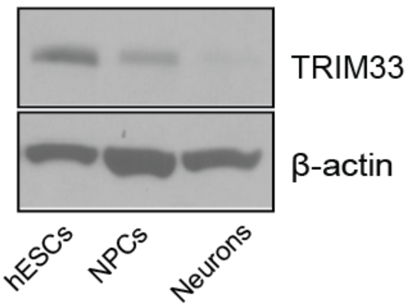

Supplementary Figure 2. Analysis of TRIM33 protein levels in H9 hESCs. Western blot with antibody against TRIM33 comparing H9 hESCs with their differentiated neural (NPC) and neuronal counterparts. ß-actin is the loading control. Although two independent experiments showed a decrease in TRIM33 levels during neuronal differentiation as we observed in our proteomics analysis, this downregulation was not consistent in other neural differentiation experiments (please see **Fig. 1a**).

Supplementary Figure 3

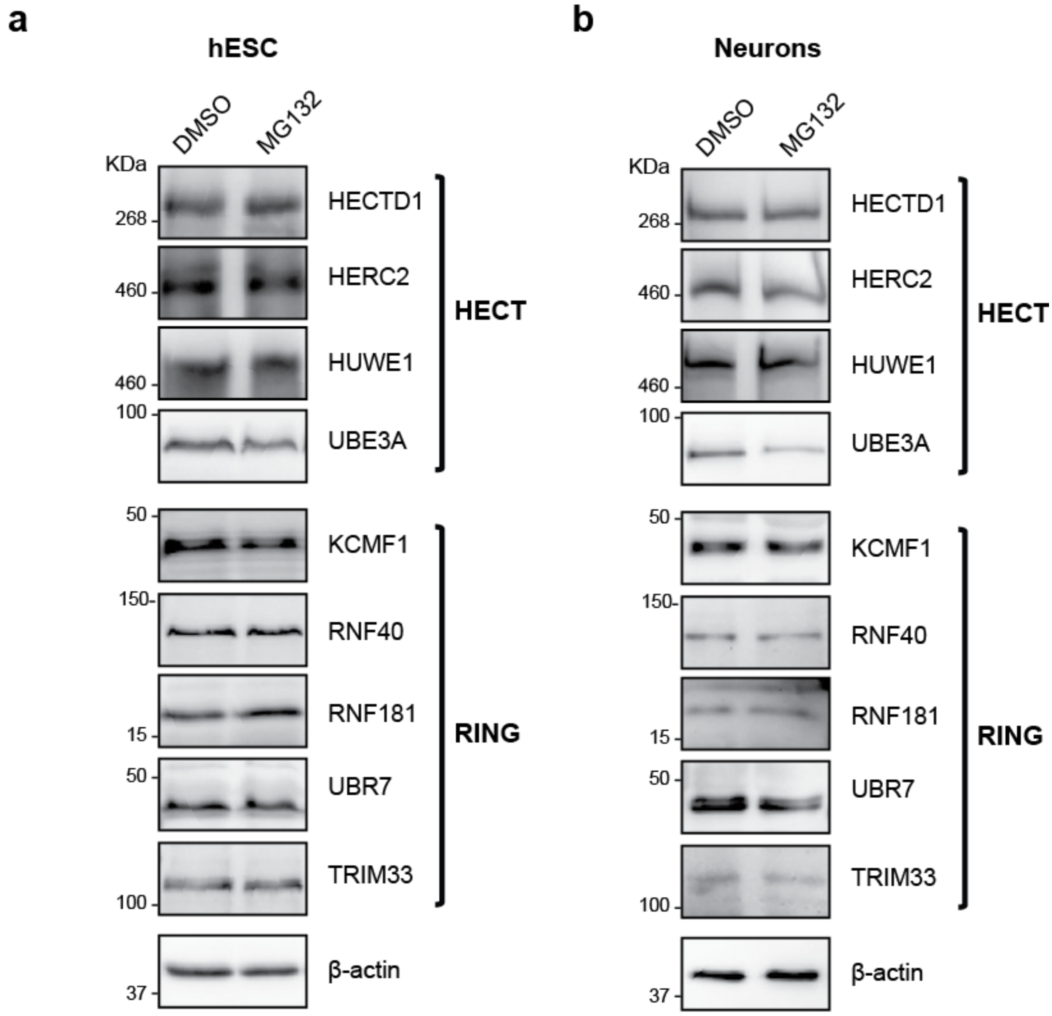

Supplementary Figure 3. Proteasome inhibition does not change protein levels of the tested E3 ligases. Western Blot analysis with antibodies against the indicated E3 enzymes of (**a**) H9 hESCs and (**b**) terminally differentiated neurons upon proteasome inhibition. Cells were treated with 1 µM MG132 for 16 h. ß-actin is the loading control. The images are representative of at least two independent experiments. All cropped blots were run under the same experimental conditions.

Supplementary Figure 4

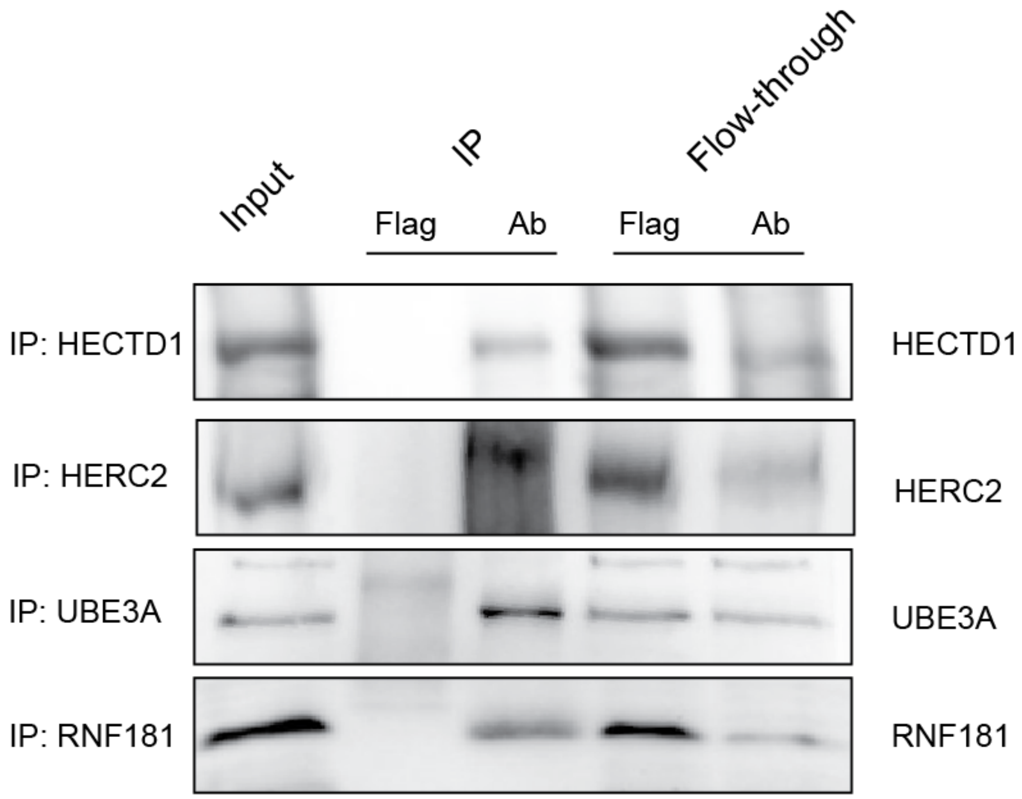

Supplementary Figure 4. Flow-through controls indicate significant immunoprecipitation of endogenous E3 enzymes. Co-immunoprecipitation (co-IP) with HERC2, HECTD1, UBE3A, RNF181 and FLAG antibodies from H9 hESCs followed by western blot against the respective E3 ubiquitin ligases. We loaded the same amount of total input and flow-through (8% of the sample) for direct comparison. We loaded 20% of total immunoprecipitated sample. The images are representative of two independent experiments.

Supplementary Figure 5

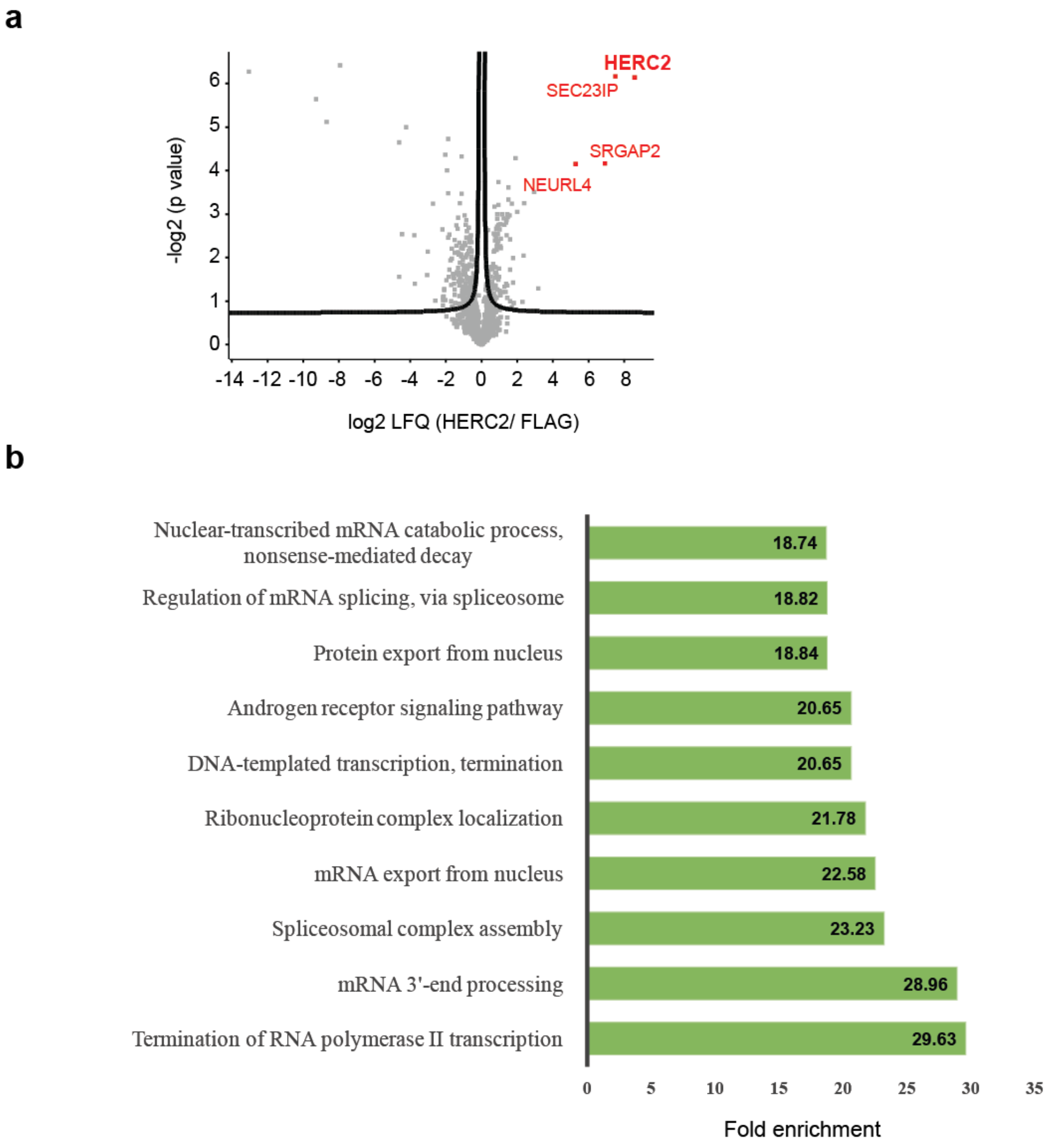

Supplementary Figure 5. Significant enriched proteins upon co-immunoprecipitation with HERC2 antibody under standard conditions (no RNase treatment). (**a**) Volcano plot of the interactome of HERC2 in H9 hESCs under standard immunoprecipitation conditions (n= 4). Graph represents the –log (p-value) of a two-tailed *t*-test plotted against the log2 ratio of protein label-free quantification (LFQ) values from co-IP experiments with HERC2 antibody compared to control co-IP with FLAG antibody. Red colored dots indicate the most enriched interacting proteins after correction for multiple testing (FDR <0.2, s0=0.1). (**b**) Bar graph representing the top GOBPs of HECTD1 interactome (P<0.05).

Supplementary Figure 6

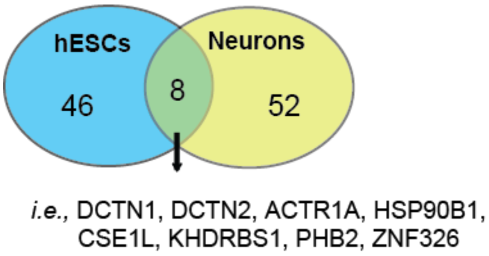

Supplementary Figure 6. Venn diagram represents total number and common significant interactors in hESCs and their differentiated neuronal counterparts (FDR <0.2 was considered significant, hESCs (n= 4) and neurons (n= 3).

Supplementary Figure 7

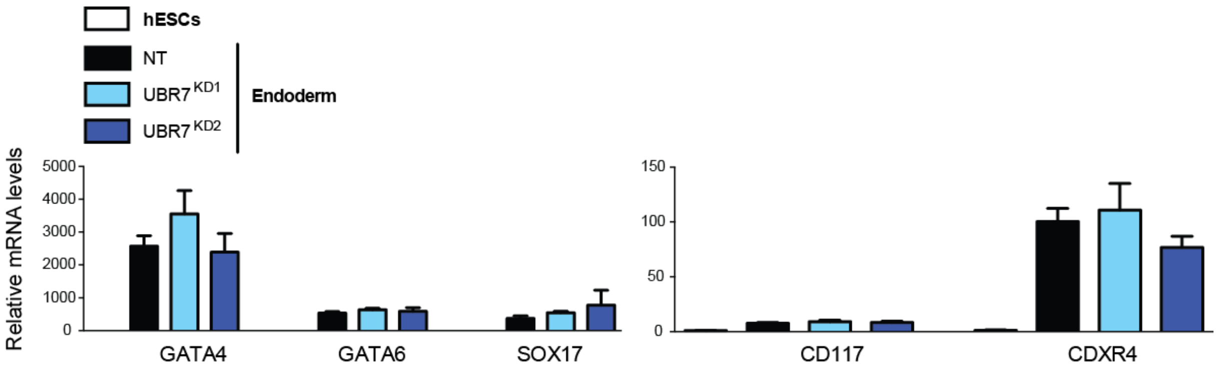

Supplementary Figure 7. Loss of UBR7 does not affect differentiation of hESCs into definitive endoderm. Real-time PCR analysis of endodermal markers in UBR7 KD H9 hESCs after definitive endodermal differentiation. Graphs (relative expression to NT) represent the mean ± s.e.m. of two independent experiments. Statistical comparisons were made by Student's *t*-test for unpaired samples.

Supplementary Figure 8

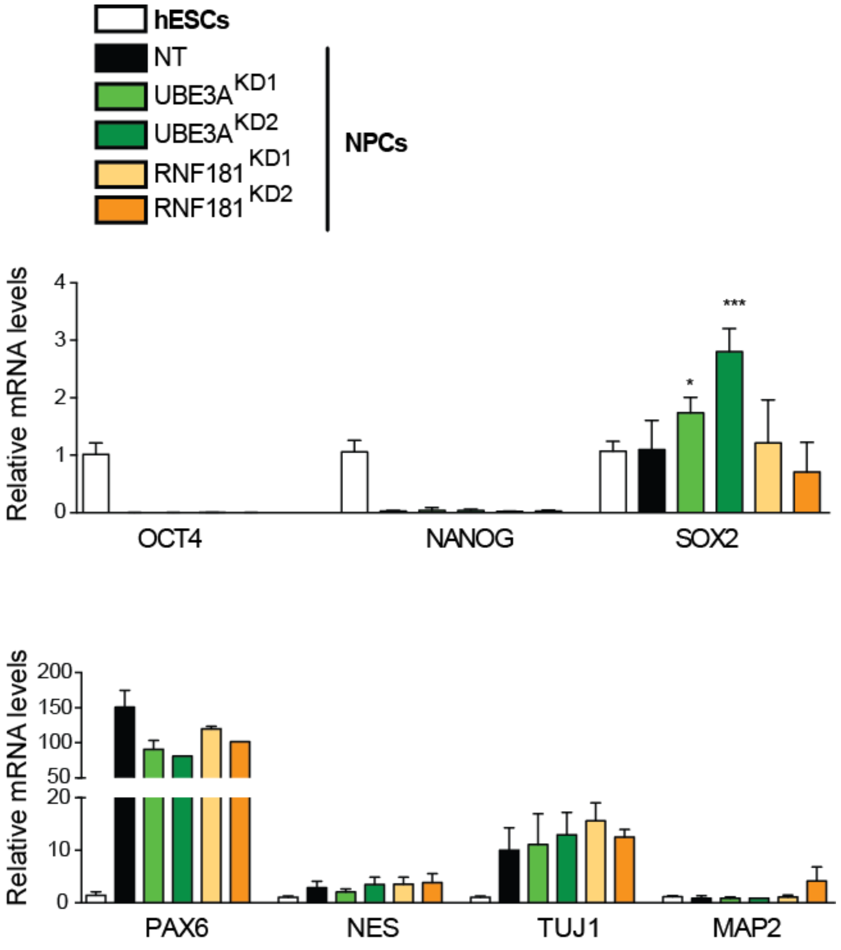

Supplementary Figure 8. Analysis of neural markers upon differentiation of UBE3A and RNF181 KD hESCs. Real-time PCR analysis of pluripotency (upper panel) and neuroectodermal markers (lower panel) in UBE3A and RNF181 KD H9 hESCs after 10 days of neural differentiation. Graphs (relative expression to NT) represent the mean ± s.e.m. of two independent experiments. Statistical comparisons were made by Student's t-test for unpaired samples. P-value: *P<0.05, ***P<0.001.

Supplementary Figure 9

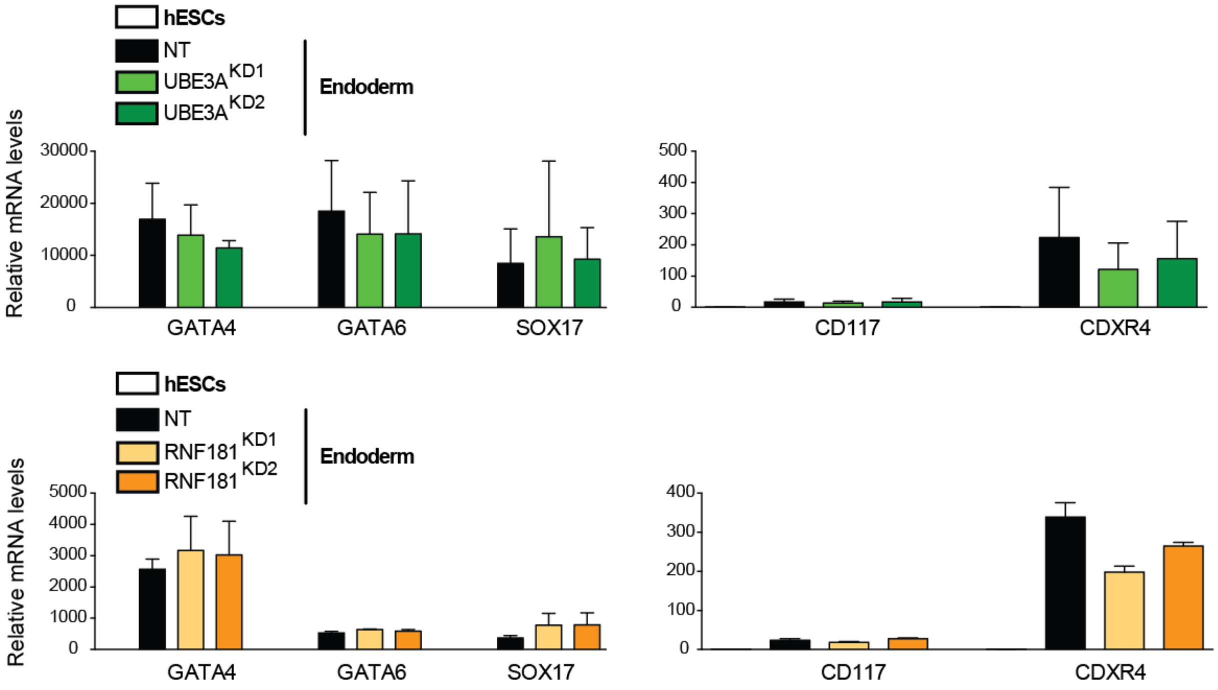

Supplementary Figure 9. Loss of UBE3A and RNF181 does not affect differentiation of hESCs into definitive endoderm. Real-time PCR analysis of endodermal markers in UBE3A (upper panel) and RNF181 (lower panel) KD H9 hESCs after definitive endodermal differentiation. Graphs (relative expression to NT) represent the mean ± s.e.m. of two independent experiments. Statistical comparisons were made by Student's *t*-test for unpaired samples.

Supplementary Figure 10

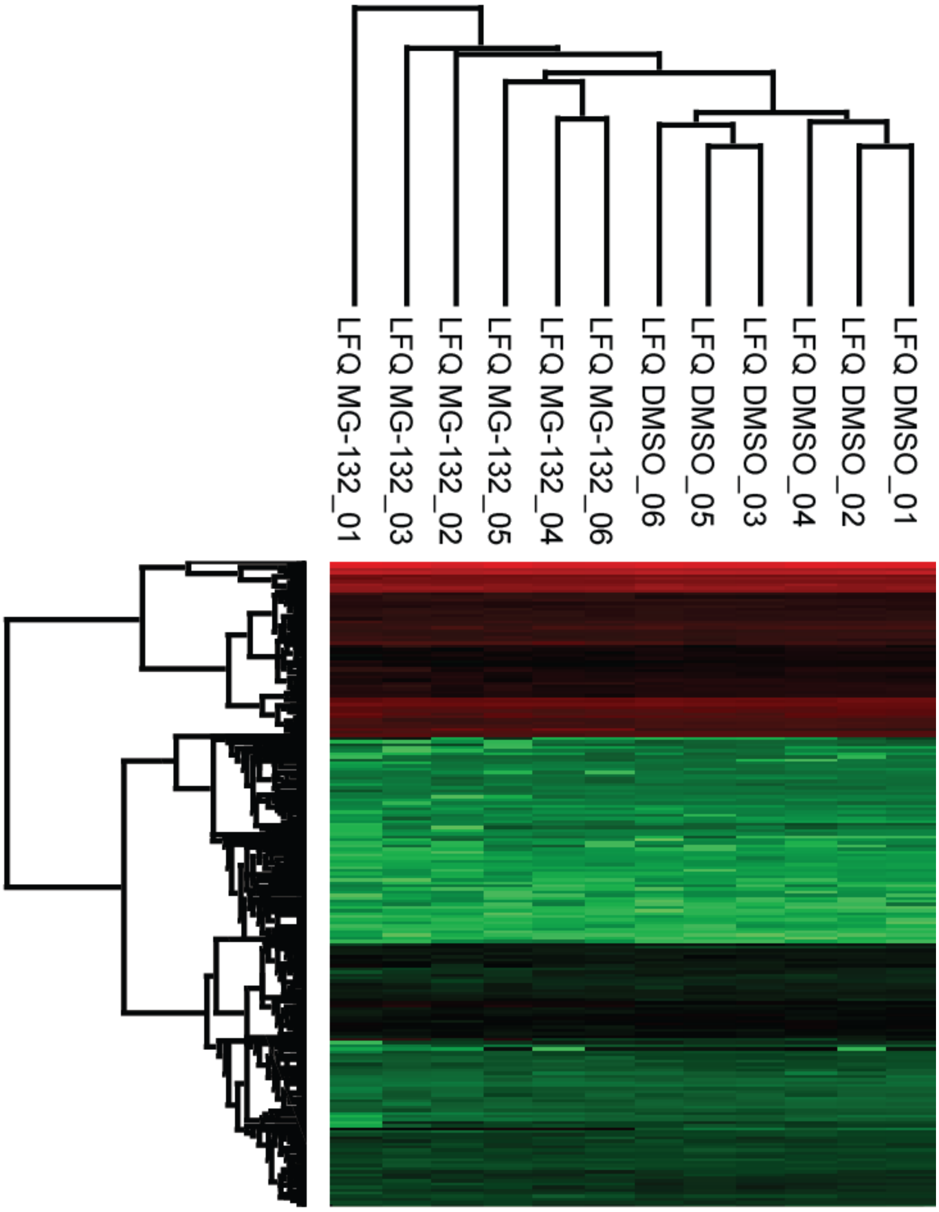

Supplementary Figure 10. Label-free quantification (LFQ) of 2735 proteins reveals partial separation of MG-132-treated H9 hESCs and control DMSO-treated H9 hESCs (n= 6). Proteins (rows) and samples (columns) are clustered according to euclidean distance (red= low, green= high abundance). Proteasome inhibitor: 1 µM MG-132 for 24 h.

Supplementary Figure 11

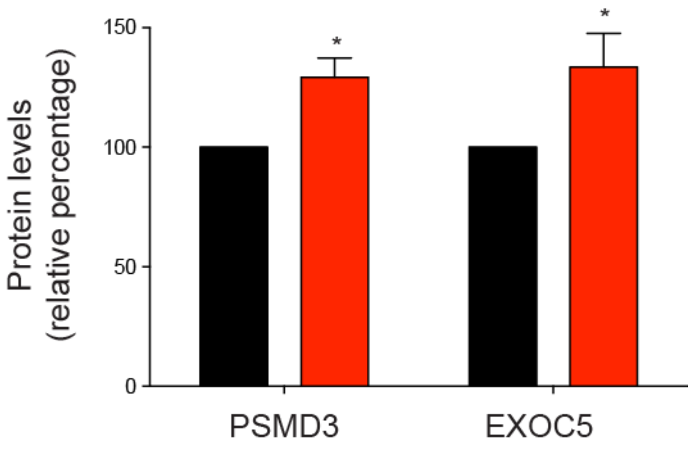

Supplementary Figure 11. Proteasome inhibition results in increased levels of PSMD3 and EXOC5. Quantification of the western blots presented in Figure 8e. The graph represents the relative percentage values to DMSO-treated H9 of PSMD3 and EXOC5 corrected for β-actin loading control (mean ± s.e.m. of three independent experiments). Statistical comparisons were made by Student's *t*-test for unpaired samples. P-value: *P<0.05.

Supplementary Figure 12

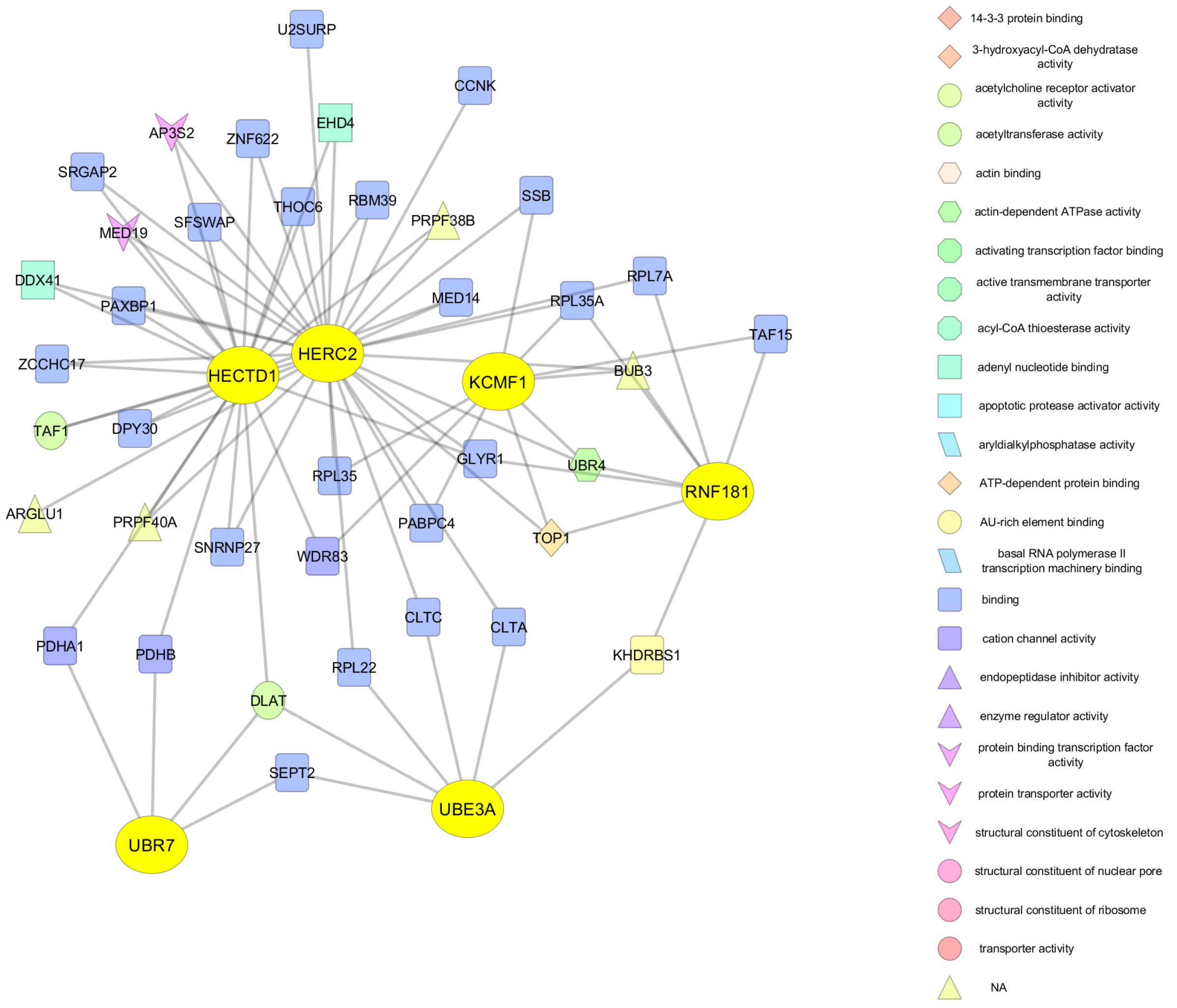

Supplementary Figure 12. Schematic network showing proteins that interact with at least two of the tested E3 enzymes.

Supplementary Figure 13

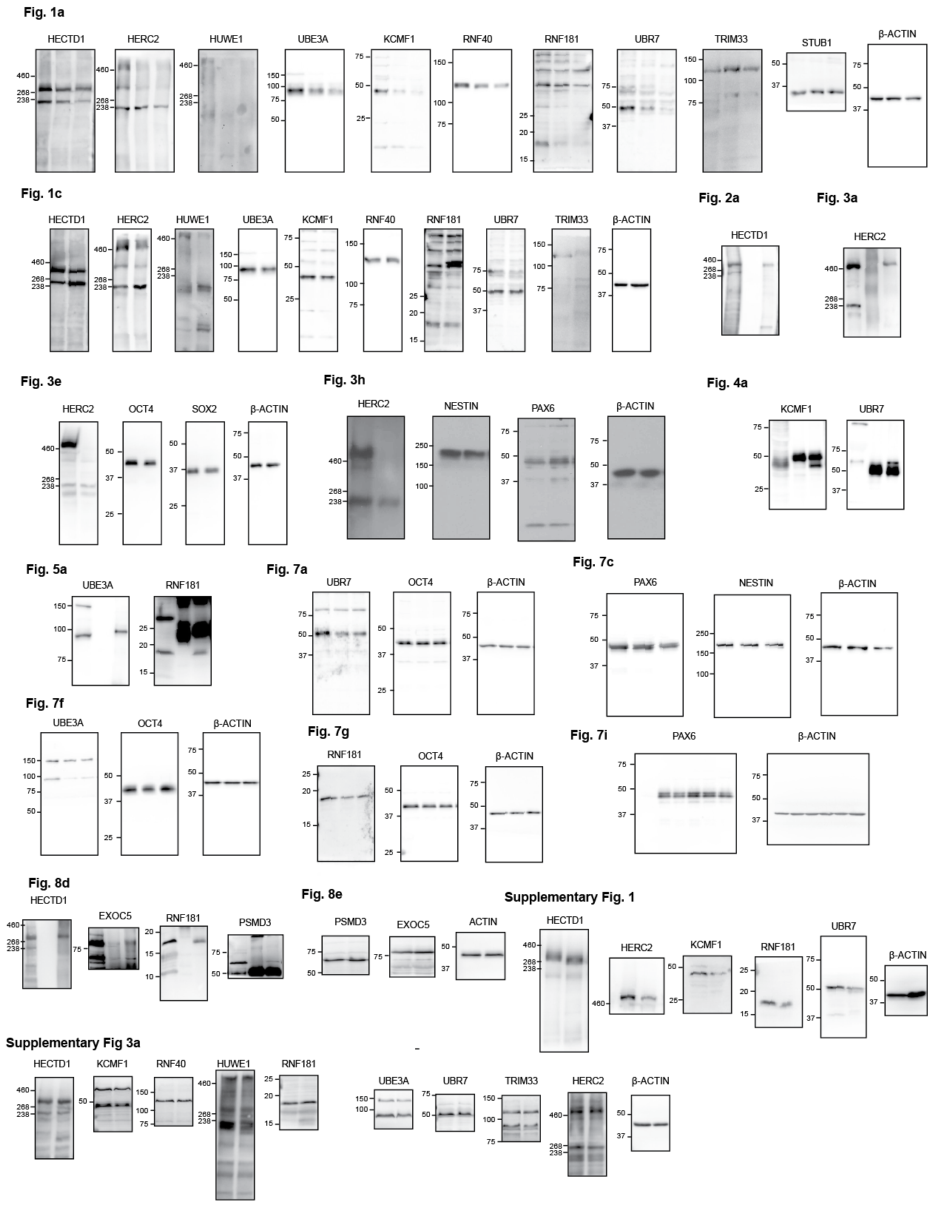

Supplementary Figure 13. Uncropped images of western blots experiments. Uncropped images are presented with molecular weight ladders.

Protein names	Domain	hESCs vs Neurons (fold enrichment)	-Log p-value	q-value	Differences supported by Western blot
FBXO22	RING	1.85	5.43	0	Not tested
KLHL4	RING	2.22	5.69	0	Not tested
TRIM24	RING	1.42	8.01	0	Not tested
TRIM28	RING	1.26	11.20	0	Not tested
TRIP12	HECT	2.08	7.,20	0	Not tested
UBR5	HECT	2.98	6.99	0	Not tested
IVNS1ABP	RING	3.20	4.01	0.0000124	Not tested
KLHL7	RING	1.72	4.26	0.0000136	Not tested
RBX1	RING	1.01	5.54	0.0000138	Not tested
TRIM33	RING	1.31	4.81	0.0000139	NO
PPIL2	RING	1.96	4.28	0.0000145	Not tested
UHRF1	RING	2.42	3.71	0.0000180	Not tested
SKP2	RING	1.77	3.86	0.0000242	Not tested
KEAP1	RING	2.69	3.31	0.0000380	Not tested
ARIH2	RING	0.79	4.94	0.0000506	Not tested
UBR7	RING	2.28	3.08	0.0000536	YES
RBBP6	RING	2.81	2.66	0.0000964	Not tested
ZNF598	RING	1.55	2.80	0.000143	Not tested
UBE4B	RING	0.89	3.33	0.000191	Not tested
TRIM65	RING	1.46	2.51	0.000283	Not tested
UBE3A	HECT	0.63	3.64	0.000345	YES
RNF2	RING	0.67	3.07	0.000645	Not tested
NOSIP	RING	0.69	2.96	0.000680	Not tested
TRIM56	RING	1.21	1.96	0.00139	Not tested
RNF40	RING	0.43	3.48	0.00166	YES
RNF20	RING	0.38	3.37	0.00285	Not tested
HERC2	HECT	0.79	1.91	0.00288	YES
RNF181	RING	1.53	1.53	0.00307	YES
HECTD1	HECT	0.55	2.61	0.00367	YES
KCMF1	RING	0.73	1.83	0.00379	YES
RNF138	RING	1.15	0.77	0.0468	Not tested
KCTD15	RING	0.59	0.79	0.0645	Not tested
BRAP	RING	0.78	0.71	0.0702	Not tested
TRAF2	RING	0.25	1.08	0.0934	Not tested
HUWE1	HECT	0.12	2.45	0.162	YES

Supplementary Table 1. List of E3 ubiquitin ligases increased in hESCs compared with their neuronal counterparts. Statistical comparisons were made by Student's t-test (n= 6). False Discovery Rate (FDR) adjusted p-value (q-value) <0.2 was considered significant. Up-regulated E3s in hESCs are ranked according to the q-value.

Primers for Real Time PCR used in this study

Gene	Primer sequence
HERC2 F	GCCTTACACGCAGCCATTACT
HERC2 R	TTCCTTCTGTGCTTCTTTTTTGG
UBE3A F	GACTCAAAGTTAGACGTGACCATATCA
UBE3A R	CTTCAAGTCTGCAGGATTTTCCA
UBR7 F	TGCCGGCTCTAGTTCTGAATC
UBR7 R	TTCTGCGTTGAGGCTTTCATT
KCMF1 F	GCCCCTAGAGATTTAGATGAATCG
KCMF1 R	CCCGGCCAGGGTGAAA
RNF181 F	CCTTGCCATCACCTTTTCCA
RNF181 R	GGACAGGAATTTGTCTTGCTTAGC
HECTD1 F	CAACAATTGTAAGTCTGCTCTCAACA
HECTD1 R	TCACCCTGCAATGCACTTTC
TRIM33 F	CACAGCCTACAAGCACCATGA
TRIM33 R	TGTGAGAATTGGATAAACCTGATGA
RNF40 F	CTCCCACCTGCGACACATC
RNF40 R	CCTCTGTGCGTAGCTTCTTCTG
HUWE1 F	CACAAGAAGGAGAGGAAATGGAA
HUWE1 R	AGTGCTGCTCGTTGTGGAATAC
GAPDH F	GCACCGTCAAGGCTGAGAAC
GAPDH R	GGATCTCGCTCCTGGAAGATG
ACTIN F	CTGGCACCCAGCACAATG
ACTIN R	CCGATCCACACGGAGTACTTG
OCT4 F	GGAGGAAGCTGACAACAATGAAA
OCT4 R	GGCCTGCACGAGGGTTT
NANOG F	AAATCTAAGAGGTGGCAGAAAAACA
NANOG R	GCCTTCTGCGTCACACCATT
SOX 2 F	TGCGAGCGCTGCACAT
SOX 2 R	TCATGAGCGTCTTGGTTTTCC
DPPA4 F	CTGGTGCCAACAATTGAAGCT
DPPA4 R	AGGCACACAGGCGCTTATATG

MSX1 F	CTCCGCAAACACAAGACGAAC
MSX1 R	CACATGGGCCGTGTAGAGTC
GATA4 F	TCCGTGTCCCAGACGTTCTC
GATA4 R	GAGAGGACAGGGTGGATGGA
GATA6 F	AGCGCGTGCCTTCATCA
GATA6 R	GTGGTAGTTGTGGTGTGACAGTTG
ALBUMIN F	TGAGGTTGCTCATCGGTTTAAA
ALBUMIN R	GCAATCAACACCAAGGCTTTG
PAX6 F	CATACCAAGCGTGTCATCAATAAAC
PAX6 R	TGCGCCCATCTGTTGCT
NESTIN F	TGAAGGGCAATCACAACAGG
NESTIN R	TGACCCCAACATGACCTCTG
MAP2 F	AAAGAAGCTCAACATAAAGACCAGACT
MAP2 R	GTGGAGAAGGAGGCAGATTAGC
SOX1 F	GCTGACACCAGACTTGGGTT
SOX1 R	GTGCTTGGACCTGCCTTACT
TUJ1 F	GGCCAAGTTCTGGGAAGTCA
TUJ1 R	CGAGTCGCCCACGTAGTTG
SOX17 F	TGGCGCAGCAGAATCCA
SOX17 R	CGACTTGCCCAGCATCTTG
CD117 F	CCAAGGCCGACAAAAGGA
CD117 R	GGCGGGAGTCACATCTCTTTC
CDXR4 F	GGCCGACCTCCTCTTTGTC
CDXR4 R	TTGCCACGGCATCAACTG
